# Neuron-specific spinal cord translatomes reveal a neuropeptide code for mouse dorsal horn excitatory neurons

**DOI:** 10.1038/s41598-021-84667-y

**Published:** 2021-03-04

**Authors:** Rebecca Rani Das Gupta, Louis Scheurer, Pawel Pelczar, Hendrik Wildner, Hanns Ulrich Zeilhofer

**Affiliations:** 1 Institute of Pharmacology and Toxicology, University of Zurich, Winterthurerstrasse 190, 8057 Zurich, Switzerland; 2 Institute of Pharmaceutical Sciences, Swiss Federal Institute of Technology (ETH) Zurich, Vladimir-Prelog-Weg 1-5/10, 8090 Zurich, Switzerland; 3 Center for Transgenic Models, University of Basel, 4001 Basel, Switzerland

**Keywords:** Neuroscience, Cellular neuroscience, Genetics of the nervous system, Molecular neuroscience, Neural circuits, Sensory processing, Somatosensory system

## Abstract

The spinal dorsal horn harbors a sophisticated and heterogeneous network of excitatory and inhibitory neurons that process peripheral signals encoding different sensory modalities. Although it has long been recognized that this network is crucial both for the separation and the integration of sensory signals of different modalities, a systematic unbiased approach to the use of specific neuromodulatory systems is still missing. Here, we have used the translating ribosome affinity purification (TRAP) technique to map the translatomes of excitatory glutamatergic (vGluT2^+^) and inhibitory GABA and/or glycinergic (vGAT^+^ or Gad67^+^) neurons of the mouse spinal cord. Our analyses demonstrate that inhibitory and excitatory neurons are not only set apart, as expected, by the expression of genes related to the production, release or re-uptake of their principal neurotransmitters and by genes encoding for transcription factors, but also by a differential engagement of neuromodulator, especially neuropeptide, signaling pathways. Subsequent multiplex in situ hybridization revealed eleven neuropeptide genes that are strongly enriched in excitatory dorsal horn neurons and display largely non-overlapping expression patterns closely adhering to the laminar and presumably also functional organization of the spinal cord grey matter.

## Introduction

The ability to sense and discriminate different noxious and innocuous somatosensory stimuli is essential for all higher animals and humans in order to react adequately to external stimuli and internal conditions^1,2^. The spinal dorsal horn, i.e., the sensory part of the spinal cord, constitutes a key element in this process. It receives somatosensory signals from peripheral neurons and processes these signals together with other inputs descending from supraspinal sites in a complex network of inhibitory and excitatory interneurons before relaying them via projection neurons to supraspinal centers^3^. Projection neurons make up less than 10% of all dorsal horn neurons, while more than 90% of the neuronal population are interneurons of which, between 60 and 70% are excitatory glutamatergic neurons, and the rest is inhibitory (GABA and/or glycinergic)^3,4^. Besides these principal neurotransmitters, many neurons of the spinal dorsal horn use additional signaling molecules, especially neuropeptides, for neuronal communication, which together with the structural connectivity set the basis for modality specific sensory processing.

The spinal cord is organized in a laminar fashion, which has initially been proposed on the basis of differences in cell density and morphology^5,6^ but has later been shown to also reflect a functional organization. This becomes obvious especially from the lamina specific innervation pattern by the different types of peripheral sensory neurons: unmyelinated C fibers, which mainly carry noxious and thermal information, terminate in the superficial dorsal horn (laminae I–II), while thickly myelinated Aβ fibers, which convey innocuous signals including touch and proprioceptive information, terminate in the deep dorsal horn (laminae III–V)^3,7,8^. A laminar organization of neuronal function is also supported by gene expression patterns that follow laminar patterns^9–13^ and a recent publication indicating a topographic map for spinal sensorimotor reflexes^14^. Furthermore, optogenetic and chemogenetic experiments support a modality-specific processing by distinct genetically defined neuron populations^8,15–17^. However, the basis of this modality specific processing beyond structural connectivity is only incompletely understood.

In the present study, we performed a genome-wide unbiased search for genes involved in neuronal communication in excitatory or inhibitory spinal cord neurons. To this end, we employed the translating ribosome affinity purification (TRAP) technology^18^ to characterize the translatomes (i.e. the whole range of mRNAs that are not only transcribed but also translated). We generated vGluT2::bacTRAP (*Slc17a6*), vGAT::bacTRAP (*Slc32a1*) and Gad67::bacTRAP (*Gad1*) mice, which express the eGFP-tagged ribosomal subunit L10a (RPL10a) under the control of the respective gene regulatory elements. Gene ontology and pathway analyses revealed that, in addition to transcription factors and genes related to the neurotransmitter phenotype of these neurons, genes encoding elements of neuropeptide signaling constitute a functionally defined class of genes that is highly enriched in excitatory spinal neurons. About 25% of the most strongly enriched genes were neuropeptides. Subsequent multiplex in situ hybridization for neuropeptide encoding genes revealed largely non-overlapping expression patterns that follow the laminar organization of the spinal cord thus suggesting a role of neuropeptide signaling in segregation of sensory modalities.

## Results

### Generation and validation of three bacTRAP mouse lines to profile gene translation in excitatory and inhibitory neurons

**Figure 1:**
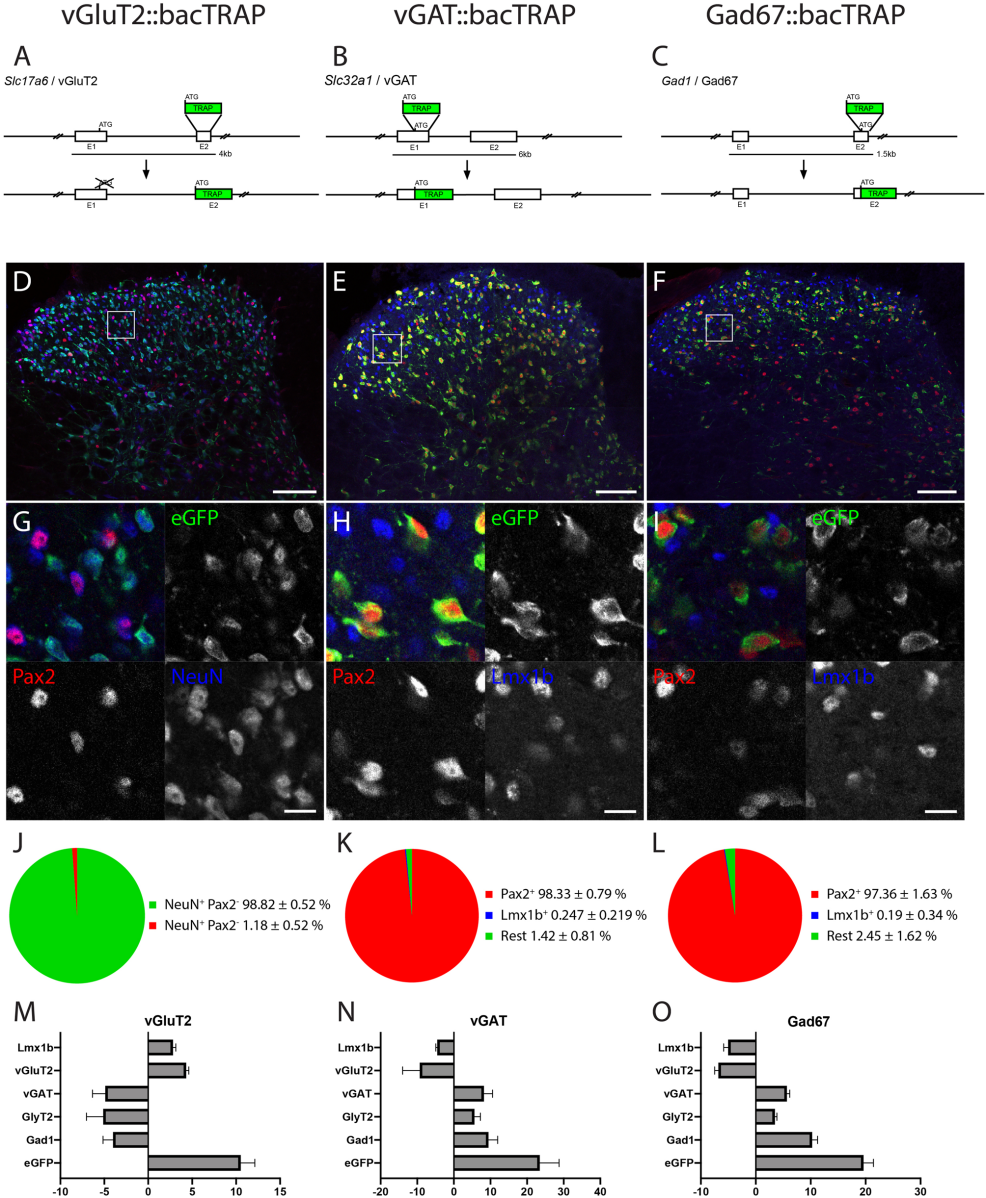
Generation and validation of three bacTRAP mouse lines, specific to excitatory and inhibitory spinal cord neurons. (**A**,**D**,**G**,**J**) vGluT2::bacTRAP line, (**B**,**E**,**H**,**K**) vGAT::bacTRAP line, (**C**,**F**,**I**,**L**) Gad67::bacTRAP line. (**A**–**C**) BAC constructs for the three mouse lines. (**D**–**I**) Immunofluorescence images of the lumbar spinal dorsal horn of the three mouse lines. Boxes in the overview images of (**D**–**F**) indicate the position of the higher magnification images in (**G**–**H**). (**D**,**G**) vGluT2::bacTRAP line with eGFP in green, Pax2 in red and NeuN in blue. (**E**,**H**) vGAT::bacTRAP mouse line with eGFP in green, Pax2 in red and Lmx1b in blue. (**F**,**I**) Gad67::bacTRAP mouse line with eGFP in green, Pax2 in red and Lmx1b in blue. (**J**) Quantification of eGFP+NeuN+ cells with and without signal for Pax2 in the vGluT2::bacTRAP line. (**K**,**L**) Quantification of eGFP + cells with signal for Pax2 or Lmx1b in (**K**) the vGAT::bacTRAP line and (**L**) the Gad67::bacTRAP line. (**M**–**O**) RT-PCR based quantification of the enrichment of selected genes comparing the polysome bound fraction to the input. (**M**) Enrichment of *eGFP* (10.5 fold ± 1.6), *Lmx1b* (2.8 fold ± 0.3) and *vGluT2* (4.3 fold ± 0.3) and depletion of *vGAT* (− 4.9 fold ± 1.5), *GlyT2* (− 5.1 fold ± 1.9) and *Gad1* (− 4-fold ± 1.2) after polysome purification from vGluT2::bacTRAP mice is depicted. (**N**) Enrichment of *eGFP* (23.4 fold ± 5.3), *vGAT* (8.2fold ± 2.4), *GlyT2* (5.6 fold ± 1.7) and *Gad1* (9.4 fold ± 2.5) and depletion of *Lmx1b* (− 4.5 fold ± 0.4) and *vGluT2* (− 9.3 ± 4.7) after polysome purification from vGAT::bacTRAP mice is depicted. (**O**) Enrichment of *eGFP* (19.6 fold ± 1.1), *vGAT* (5.625 fold ± 0.3), *GlyT2* (3.4 fold ± 0.2) and *Gad1* (10.2fold ± 0.6) and depletion of *Lmx1b* (− 5-fold ± 0.3) and *vGluT2* (− 6.8 fold ± 0.4) after polysome purification from Gad67::bacTRAP mice is depicted. (**D**–**G**) Scale bar = 100 μm. (**G**,**H**) Scale bar 5 μm. (**M**–**O**) error bar = SEM.

In this study, we set out to investigate the neuron type- and potentially lamina-specific use of neuromodulator systems in dorsal horn neurons. As part of an unbiased genome wide approach we analyzed the translatomes (polysome-bound mRNA) of excitatory and inhibitory neurons of the mouse spinal cord. To this end, we generated three bacTRAP mouse lines; vGluT2::bacTRAP (Tg(Slc17a6-RPL10a-eGFP)Uze), Gad67::bacTRAP (Tg(Gad1-RPL10a-eGFP)Uze) and vGAT::bacTRAP mice (Tg(Slc32a1-RPL10a-eGFP)Uze). In these mice, the eGFP-tagged ribosomal protein L10a is expressed as a transgene either in glutamatergic neurons (vGluT2::bacTRAP (Fig. 1A,D,G), all inhibitory neurons (GABAergic and glycinergic neurons) (vGAT::bacTRAP, Fig. 1B,E,H) or in GABAergic neurons that utilize Gad67 for GABA synthesis (Gad67::bacTRAP, Fig. 1C,F,I).

From the spinal cord of these mice, polysome-bound mRNA can be isolated by co-immunoprecipitation of mRNA bound to the eGFP-L10a tagged ribosomes. We confirmed the correct expression of the eGFP-L10a transgene in the lumbar spinal cord of the different mouse strains by immunohistochemistry. NeuN was used as a neuronal marker, Pax2 as a ubiquitous marker for spinal inhibitory^19,20^ and Lmx1b as a marker for the majority of dorsal spinal excitatory neurons (Fig. 1D–I). In the vGluT2::bacTRAP line, the eGFP-L10a expression is restricted to Pax2-negative neurons (98.8 ± 0.5% Pax2^−^ NeuN^+^; Fig. 1D,G,J), indicating its exclusive expression in excitatory spinal neurons. In the two mouse lines targeting inhibitory neurons, vGAT::bacTRAP and Gad67::bacTRAP, the expression was confined to Pax2-positive neurons (98.3 ± 0.8%, 97.4 ± 1.6%, respectively) and virtually absent from excitatory, Lmx1b-positive neurons of the dorsal horn (0.25 ± 0.22%, 0.19 ± 0.34%, for vGAT::bacTRAP and Gad67::bacTRAP mice, respectively; Fig. 1E,F,H,I,K,L). Next, we determined the fraction of *vGluT2*, *vGat* and *Gad67* mRNA expressing neurons that co-express the eGFP-L10a (TRAP) transgene in the respective TRAP driver line. To this end, we performed multiplex in situ hybridizations on lumbar spinal cord sections of Gad67::bacTRAP, vGAT::bacTRAP and vGluT2::bacTRAP mice (Fig. S1). 96.5 ± 3% of Gad67 + neurons (Fig. S1A–C), 93.8 ± 4% of vGAT neurons (Fig. S1D–F) and 95.4 ± 1.2% of vGluT2 neurons (Fig. S1G–I) expressed the TRAP transgene in the respective mouse line. To further validate the three bacTRAP lines we isolated the mRNA bound to the eGFP-L10a tagged ribosomes from each line and analyzed the enrichment of selected marker genes in the polysome pull down fraction versus the input using qPCR. In all cases, we found an enrichment of the transcript encoding the transgene (eGFP) (Fig. 1M–O). In addition, when comparing the pull down fraction versus the input fraction isolated from the vGluT2::bacTRAP line we found an enrichment of transcripts encoding excitatory markers (*Lmx1b* and *vGluT2)* and a depletion of the transcripts encoding for inhibitory markers (*vGAT*, *GlyT2* and *Gad1)* (Fig. 1O). Conversely, in the two lines expressing the TRAP construct in inhibitory neurons we found an enrichment of *vGAT, GlyT2 and Gad1* and a depletion of *Lmx1b* and *vGluT2* (Fig. 1N,M).

### General differences in gene expression in excitatory and inhibitory spinal neurons

**Figure 2:**
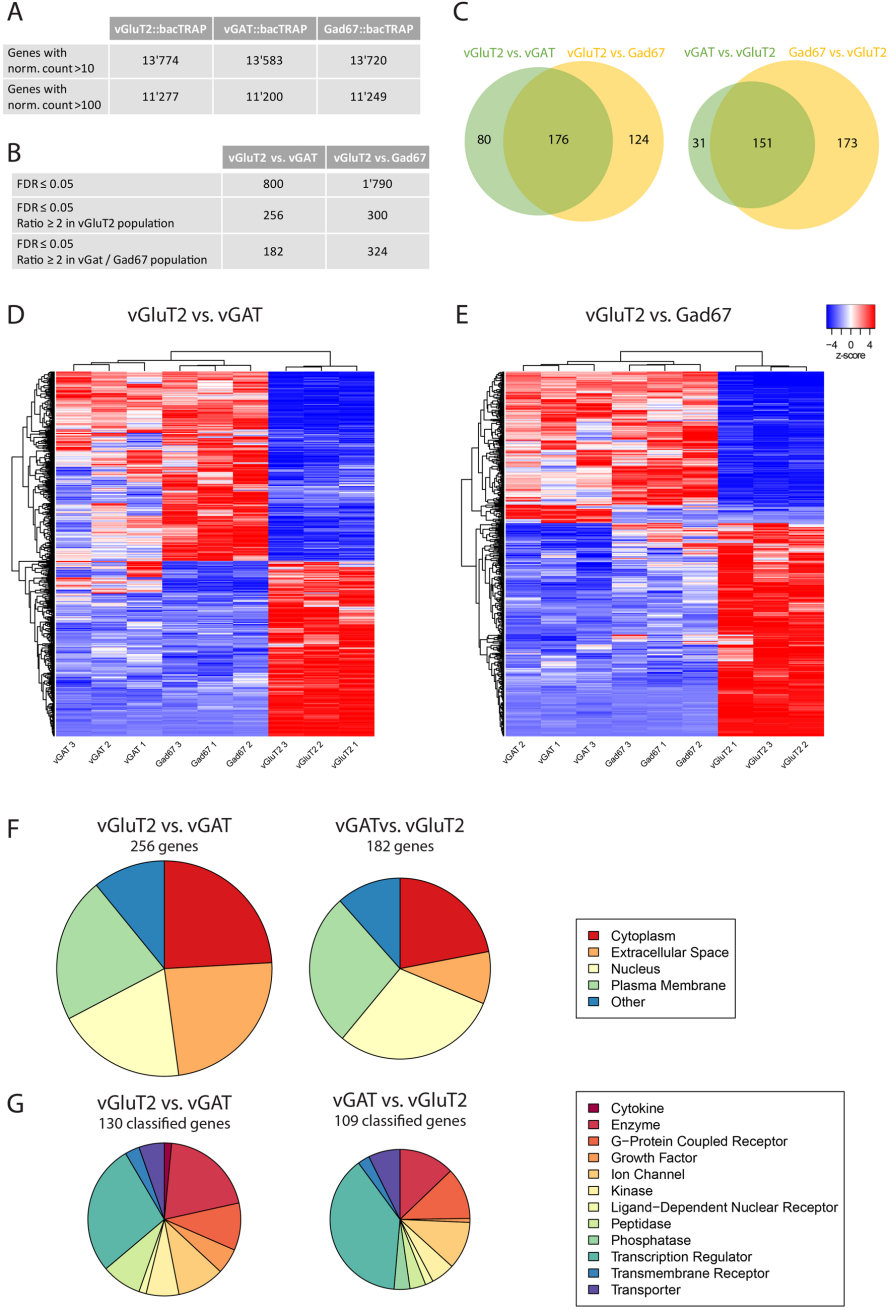
RNA-Seq metadata from sequencing of the polysomal mRNA from vGluT2::bacTRAP, vGAT::bacTRAP and Gad67::BacTRAP mice. (**A**) Number of “expressed” genes (> 10 norm. counts) and number of genes with > 100 norm. counts in the three different mouse lines. (**B**) Number of significantly enriched genes (FDR ≤ 0.05) in the DGEAs between vGluT2::bacTRAP and vGAT::bacTRAP / Gad67::bacTRAP. Number of significantly enriched genes with ratio ≥ 2 in the excitatory and inhibitory neurons of both DGEAs. (**C**) Congruence between the enriched genes of the two DGEAs: 176 genes in common for the genes enriched in vGluT2 neurons and 151 genes in common for the genes enriched in the inhibitory neurons. (**D**,**E**) Cluster analysis with the nine mRNA samples and the genes differentially expressed in (**D**) vGluT2 vs. vGAT (800 genes, FDR ≤ 0.05) and (**E**) vGluT2 vs. Gad67 (1790 genes, FDR ≤ 0.05). Color key indicated z-score with white representing zero, blue representing negative z-scores and red representing positive z-scores. (**F**) Subcellular location and (**G**) coarse function of the genes enriched in vGluT2 vs. vGAT and vGAT vs. vGluT2. Genes with unclassified function are not displayed.

Next, we used the bacTRAP mouse lines to identify key features and subtype specific markers of the two main subpopulations of spinal interneurons (i.e. excitatory or inhibitory dorsal horn neurons). To this end, we isolated and sequenced the cell-type-specific polysomal mRNA from the lumbar spinal cord of three animals from each mouse line. Out of 22,880 detectable genes, between 13,515 and 13,774 were found expressed in each mouse line (Fig. 2A, Table S1), defined by a normalized count (number of reads per gene, normalized to the total number of reads and the gene length) of > 10. Table S1 thus presents the first source of a browsable genome wide atlas of translated mRNAs in excitatory and inhibitory spinal neurons. In order to identify those genes that show a regionally non-overlapping expression pattern, we focused our analyses on genes whose regional expression was accessible by in situ hybridization. To this end, we assumed that genes with normalized counts > 100 should be reliably detectable with in situ hybridization. This threshold is three times lower than the expression of *Grpr*, a gene that we previously found expressed at low levels in a small subset of dorsal horn neurons. In all three mouse lines, approximately 11,200 genes were expressed at levels exceeding this threshold (Fig. 2A). Differential gene expression analyses (DGEAs) revealed 800 genes that were differentially expressed (with a false discovery rate (FDR) ≤ 0.05) in vGluT2::bacTRAP versus vGAT::bacTRAP mice, and 1,790 genes in vGluT2::bacTRAP versus Gad67::bacTRAP mice (Fig. 2B). The approximately two-fold lower number of significantly enriched genes in the comparison of vGluT2::bacTRAP versus vGAT::bacTRAP mice probably results from the lower expression of the eGFP-L10a transgene in the vGAT::bacTRAP line and hence a lower mRNA yield and higher variability in read numbers.

When we restricted the analysis to genes with an at least two-fold enrichment, comparison of the translatomes of *vGluT2* positive and *vGAT* positive neurons revealed 438 differentially expressed genes. Of those, 256 genes were enriched in excitatory neurons, and 182 in inhibitory neurons. When the Gad67-TRAP line was used instead of the vGAT-TRAP line, a higher number of differentially expressed genes were detected (624, of which 300 and 324 were enriched in excitatory and inhibitory neurons, respectively) (Fig. 2B). Between 70 and 80% of the differentially expressed genes identified with the vGAT-TRAP line were also found in the comparison with the Gad67-TRAP line (Fig. 2C), supporting the robustness of our approach.

Subsequent cluster analyses (^21^(http://fgcz-shiny.uzh.ch/fgcz_heatmap_app/)) over the nine different samples revealed, as expected, that the three expression analyses made for each mouse line clustered together. As expected, the next hierarchical segregation separated the inhibitory (vGAT and Gad67) from the excitatory (vGluT2) samples. Within the inhibitory cluster vGAT samples segregated from Gad67 samples (Fig. 2D,E). The cluster analysis therefore identified the vGAT^+^, Gad67^+^ and vGluT2^+^ samples as three populations with distinct translatome profiles.

To further validate our bacTRAP approach we compared our gene expression results to recently published single cell sequencing data. We chose the 50 genes that were most enriched in one of the three bacTRAP lines employed in our study to the single cell sequencing data of Haring et al.^9^ (Tables S2–S5). We found that many of our hits were also present in the single cell data (60–70% of the 50 most enriched genes). Fourteen to 20 genes identified per comparison in our study were either not detected or only at a level that was too low to assign them to a subpopulation. Among those genes that were not detected by the single cell analysis were many transcription factors such as *Vsx2*, *Lmx1b*, *Pax5*, *Barhl1* or *Phox2a* but also other genes (e.g. *Ighg3*). To verify the expression of some of these genes, we selected three for further investigation with in situ hybridization. We verified expression of *Barhl1* and *Phox2a*, which we found enriched in excitatory cells and the immunoglobulin heavy chain gene *Ighg3*, which we surprisingly found enriched in inhibitory neurons (Fig. S2).

Finally, we were interested in the correlation of the genes highly enriched in our study with the molecular groups that had been defined by Haring et al.^9^. We therefore analyzed the distribution of the 50 most enriched genes among the 30 neuronal subpopulations identified by Haring et al. (Tables S2–S5). Among the genes found enriched in excitatory neurons in our study (Tables S2, S4), three genes (*Nrn1, Cacna2d1* and *Slc17a6*) displayed expression in all 15 excitatory subtypes of Haring et al. Three other genes (*Ucn3, Cpne4, Aldh1a2*) have been attributed to only one excitatory subtype, while all other genes (44) were either attributed to more than one subtype or not reported in the single cell analysis. Among the genes enriched in inhibitory neurons (Tables S3, S5), seven genes displayed pan-inhibitory expression, seven were assigned to only one subtype and the remaining genes were assigned to more than one subtype or not reported. All genes that we found to be enriched in either inhibitory or excitatory neurons were (if detected) also exclusively or preferentially ascribed to the same category by Haring et al. (http://linnarssonlab.org/dorsalhorn). This comparison suggests first that the BAC-TRAP approach has a lower detection limit than in the single cell RNA sequencing approach and, second, that most of the subpopulation specific genes detected with single cell sequencing are not only transcribed but also translated.

### Subcellular localization and coarse function of enriched genes

To obtain a first impression what type of genes are enriched in inhibitory or excitatory spinal neurons we employed the Ingenuity Pathway Analysis (IPA, QIAGEN) tool, which assigns a unique subcellular location (“location”) and coarse function (“type(s)”) to the enriched genes (Fig. 2F,G). Twenty-four percent of the genes enriched in vGluT2 over vGAT encoded for cytoplasmic proteins, 24% for extracellular proteins, 22% for proteins in the plasma membrane, 20% were nuclear proteins and the remaining 11% were located elsewhere. Similar localizations were found when genes enriched in the inhibitory populations were analyzed (Fig. 2F). One hundred-thirty out of 256 genes enriched in vGluT2 over vGAT and 109 of 182 genes enriched in vGAT over vGluT2 could be assigned to a coarse function (Fig. 2G). The largest group of genes that distinguished inhibitory from excitatory neurons in the adult spinal cord were transcription regulators (28% and 39% of the genes enriched in vGAT and vGluT2, respectively). Two other large molecular groups present in the set of enriched genes were enzymes and G protein coupled receptors (Fig. 2G).

### Differential use of neurotransmitter signaling pathways

**Figure 3:**
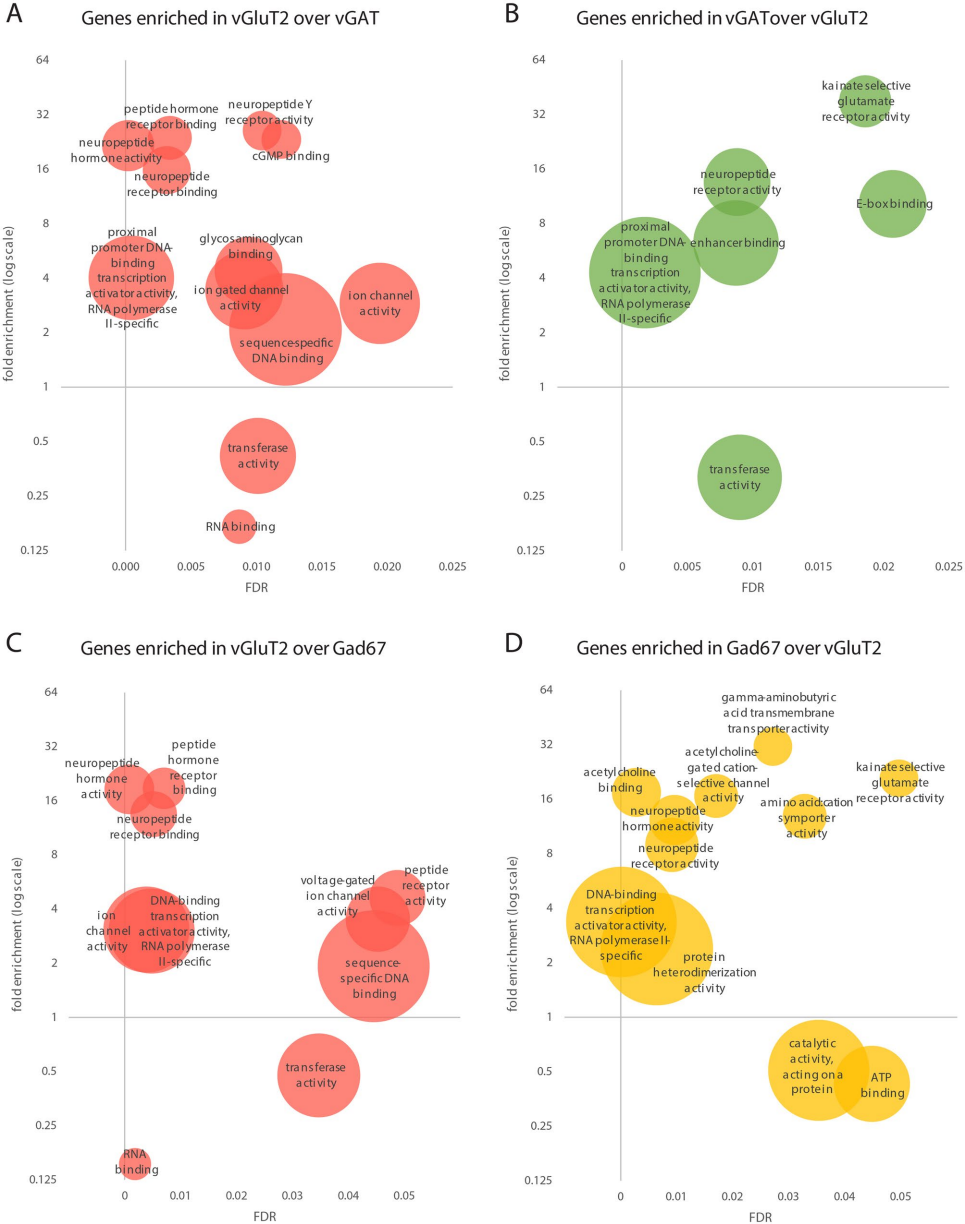
GO term overrepresentation analyses for the molecular function (MF) with the enriched genes in the comparison of vGluT2::bacTRAP to vGAT::bacTRAP and Gad67::bacTRAP. Overrepresented MF GO terms in the genes enriched in the comparison of (**A**) vGluT2 vs. vGAT, (**B**) vGAT vs. vGluT2, (**C**) vGluT2 vs. Gad67 and (**D**) Gad67 vs. vGluT2. (**A**–**D**) x axis displays false discovery rate (FDR) of the overrepresented GO term, y axis displays fold overrepresentation on a logarithmic scale and circle size indicates number of enriched genes. When several GO terms of a hierarchy are significantly overrepresented, only the most specific GO term is displayed. (**D**) For comprehensibility two MF are not displayed: “acetylcholine receptor activity”, 13.12-fold enrichment, FDR = 0.00801, no of enriched genes = 5, and “RNA polymerase II proximal promoter sequence-specific DNA binding” 2.79-fold enrichment, FDR = 0. 0.000603, no of enriched genes = 27.

**Figure 4:**
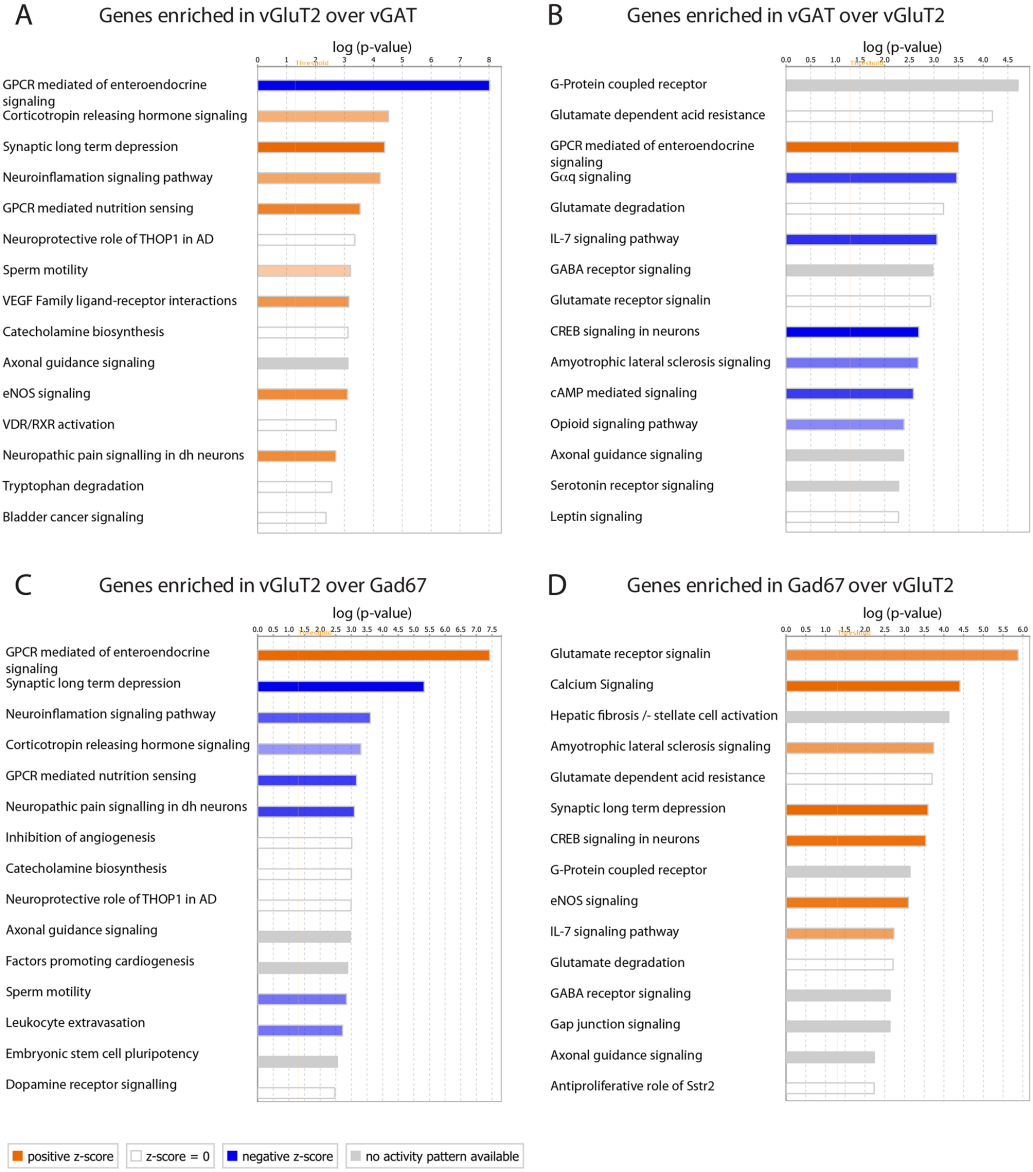
Pathway analyses with the enriched genes in the comparison of vGluT2::bacTRAP to vGAT::bacTRAP and Gad67::bacTRAP. 15 most significantly enriched pathways within the genes enriched in the comparison of (**A**) vGluT2 vs. vGAT, (**B**) vGAT vs. vGluT2, (**C**) vGluT2 vs. Gad67 and (**D**) Gad67 vs. vGluT2. (**A**–**D**) Bar length indicates significance, color key indicates activation z-score. Orange: increased activity of the pathway predicted. Blue: decreased activity of the pathway predicted. White: z-score = zero or very close to zero (overall pathway activity is neither increased nor decreased). Gray: no prediction on pathway activity possible.

We next drew our attention to the differential expression of genes involved in neuronal communication. As expected, we found a strong enrichment of genes encoding genetic markers of glutamatergic, GABAergic or glycinergic neurons (e.g. *vGluT2* (*Slc17a6*) and *vGluT3* (*Slc17a8*) in excitatory neurons, *vGAT* (*Slc32a1*), Gad67 (*Gad1*), *Gad65* (*Gad2*), *GAT1* (*Slc6a1*) and *GlyT2* (*Slc6a5*) in inhibitory neurons) (Table S1, S2–S5). However, whether other neurotransmitters such as serotonin or acetylcholine, which are released in the spinal cord, engage excitatory and inhibitory neurons differentially is less clear. We used two different pathway analysis tools, PANTHER GO-term pathway analysis^22,23^ (Fig. 3 A-D) and Ingenuity pathway analysis^24^ (QIAGEN Inc., https://www.qiagenbioinformatics.com/products/ingenuitypathway-analysis) (Fig. 4A–D) to identify interneuronal signaling pathways that are enriched in either inhibitory or excitatory spinal neurons. Pathway analysis tools differ in the algorithms and databases they rely on^22–25^. We used these two different pathway analysis tools to obtain a more comprehensive overview of signaling pathways that are enriched in either of the studied populations.

PANTHER GO-term analysis (Fig. 3A–D) indicated that *Kainate selective glutamate receptor activity* was overrepresented in the inhibitory populations (38-fold in vGAT versus vGluT2, FDR = 0.019, 21-fold in Gad67 versus vGluT2, FDR = 0.0497), mostly due to an enrichment of Grik1-3 expression in inhibitory neurons (Tables S1, S6). In addition, terms describing acetylcholine signaling related molecular function (13–17-fold, FDR = 0.0029–0.0171) and *Gamma-aminobutyric acid transmembrane transporter activity* (31.5-fold, FDR = 0.027) were overrepresented in inhibitory neurons.

The Ingenuity “pathway analysis” tool confirmed some the results of the GO term analyses but also identified additional receptor signaling pathways overrepresented in the different neuron populations (Fig. 4A–D). *Glutamate* and *GABA receptor signaling* as well as s*erotonin receptor signaling* were overrepresented in inhibitory neurons (Fig. 4B,D). As expected, the enrichment of GABAergic signaling was mainly due to genes involved in the production (*Gad1*, *Gad2*) and transport (*vGAT*, *Gat1*) of GABA (Tables S1, S2–S5), while GABA receptors showed relatively similar expression levels in the different populations (Table S8). Overrepresentation of Glutamate receptor signaling was due to the aforementioned enrichment of kainate receptors as well as to an enrichment of AMPA (*Gria1*), NMDA (*Grin2c*), and metabotropic (*Grm2*, *Grm3*) glutamate receptor subunits. Overrepresentation of serotonin receptor signaling (Fig. 4B) was due to the enrichment of serotonin receptors (*Htr1a*, *Htr1d*, *Htr3a*) in inhibitory neurons. Serotonin receptor 3a (*Htr3a*), encoding ionotropic serotonin receptors, was highly enriched in inhibitory spinal interneurons (Table S13) and displayed an expression pattern that was restricted to a population in the deep dorsal horn (Fig. S3). Tables S6–S15 illustrate relative expression levels and enrichment of glutamate, GABA, glycine, acetylcholine, serotonin, adrenergic, dopaminergic and histamine receptors. In summary, several receptor-signaling pathways are enriched within the differentially expressed genes, indicating that the respective neurotransmitter signaling pathways may engage inhibitory and excitatory spinal neurons differentially.

### A neuropeptide code for glutamatergic spinal dorsal horn neurons

**Figure 5:**
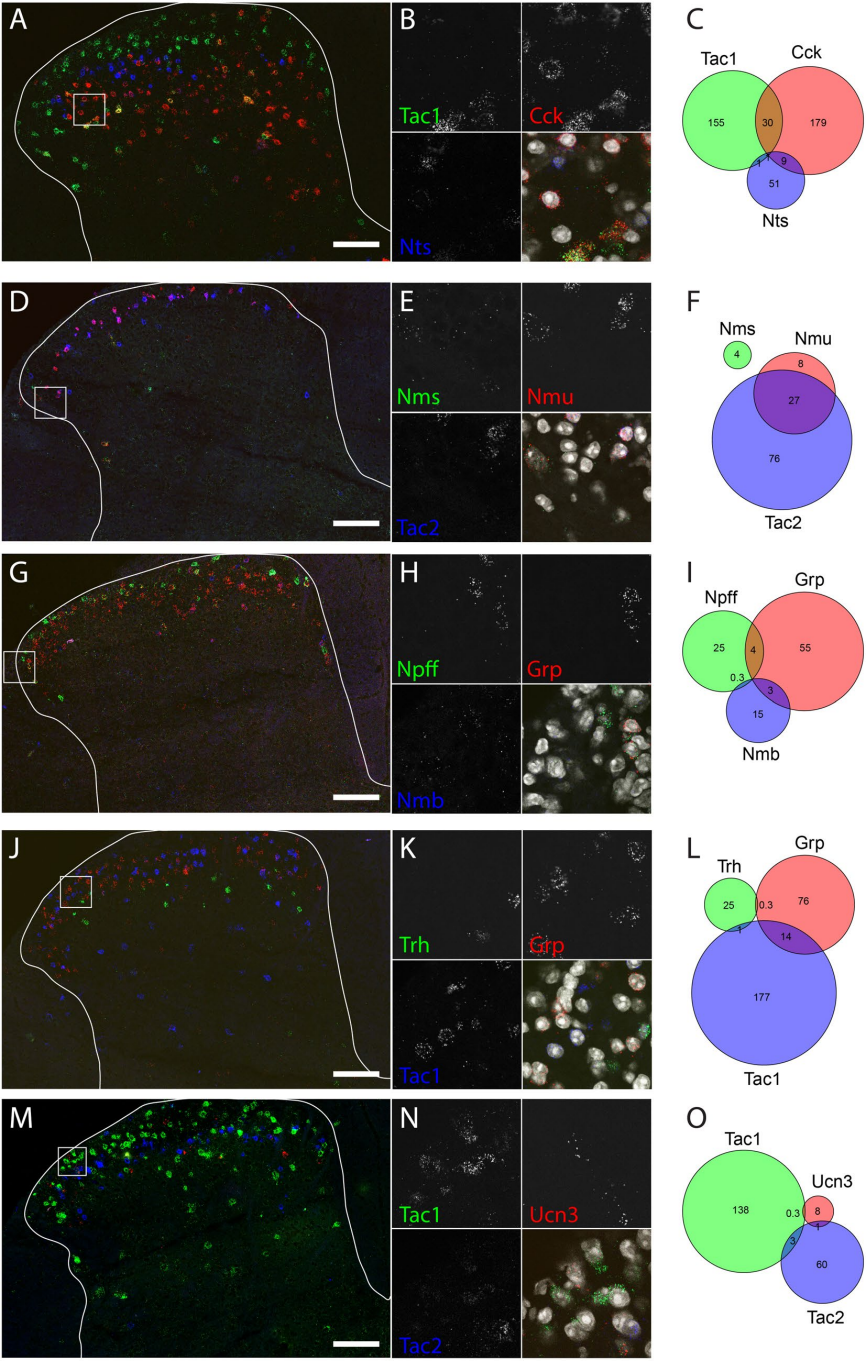
Multiplex in situ hybridization shows co-expression of eleven neuropeptides. Five combinations of three neuropeptides each: (**A**–**C**) *Tac1*, *Cck* and *Nts*; (**D**–**F**) *Nms*, *Nmu* and *Tac2*; (**G**–**I**) *Npff*, *Grp* and *Nmb*; (**J**–**L**) *Trh*, *Grp* and *Tac1*; (**M**–**O**): *Tac1*, *Ucn3* and *Tac2* in green, red and blue, respectively. (**A**,**D**,**G**,**J**,**M**) Overview images over the lumbar dorsal horn. The outline of the gray matter is indicated. Scale bar 100 μm. Box indicates position of the higher magnification image in (**B**,**E**,**H**,**K**,**N**). (**C**,**F**,**I**,**L**,**O**) Number of cells per animal (two hemi-sections) that (co-)express the three neuropeptides. Circle and intersection sizes are approximately proportional to the cell number they represent.

**Figure 6:**
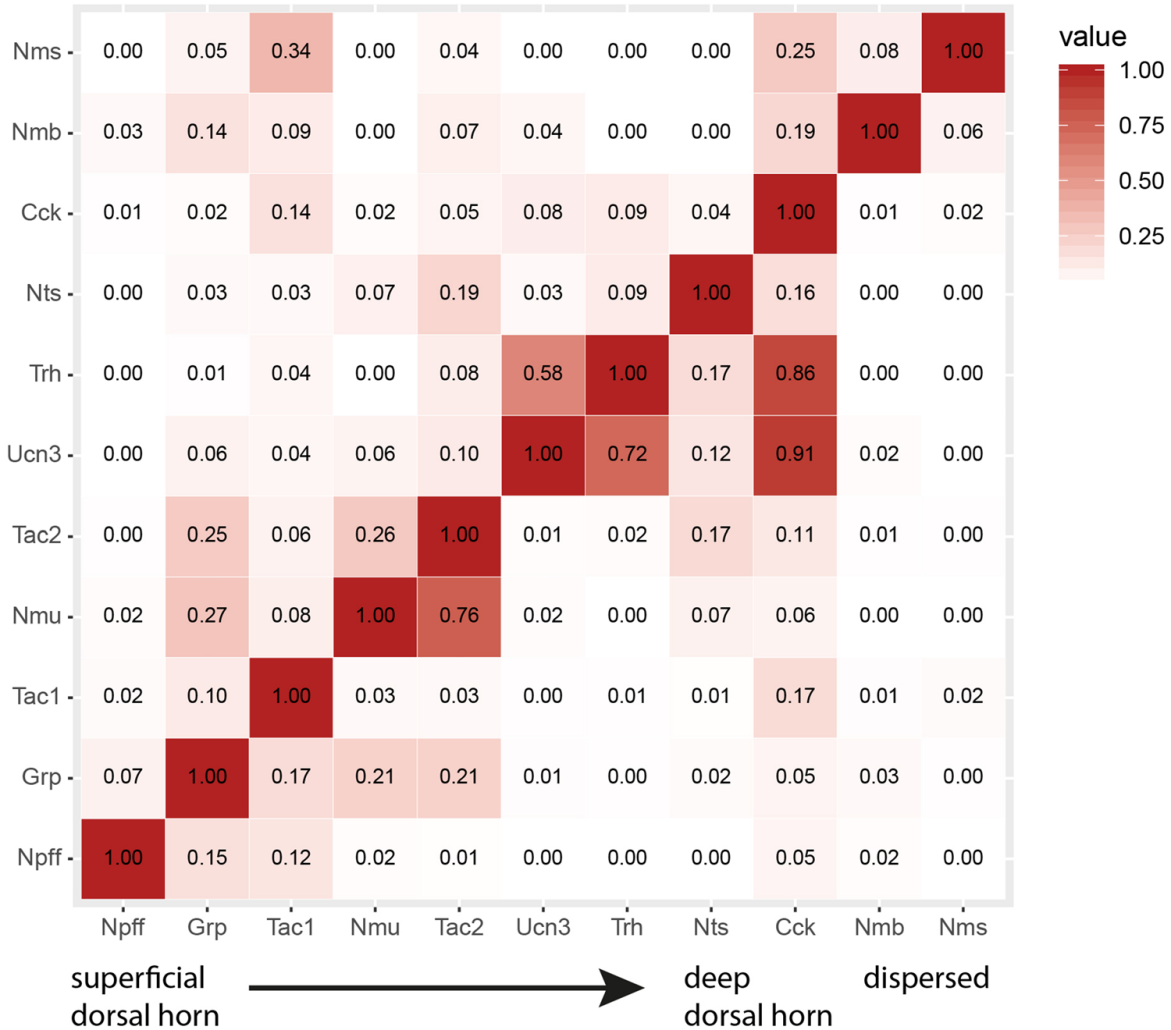
Co-expression matrix of eleven neuropeptides expressed in excitatory neurons. Co-expression within cells expressing a neuropeptide clearly above background levels (> 20 dots/cell). Depicted is the fraction of cells expressing the neuropeptide on the left, that also express the neuropeptide at the bottom. For example, 0% of the Nms cells, also express Npff. Color key indicates co-expression with light colors representing low co-expression and dark values representing high co-expression.

Neuropeptide signaling related terms (e.g. *neuropeptide hormone activity*, *neuropeptide receptor activity* and *neuropeptide receptor binding*, 4.6–26.3-fold overrepresented as compared to all expressed genes, FDR between 0.00016 and 0.0488) were highly overrepresented among the molecular function terms. These signaling terms were predominantly enriched in excitatory neurons (Fig. 3A,C). A comparison between the translatomes of the vGluT2 and Gad67 neuron populations demonstrated that 12 of the 50 genes most strongly enriched in excitatory neurons (Tables S2–S5) encoded for neuropeptides: cholecystokinin (*Cck,* 13.5-fold enriched in vGluT2 vs vGAT), gastrin releasing peptide (*Grp,* 8.6-fold enriched in vGluT2 vs vGAT), neuromedin B (*Nmb,* 18.8*-*fold enriched in vGluT2 vs vGAT), neuromedin S (*Nms,* 35.0-fold enriched in vGluT2 vs vGAT), neuromedin U (*Nmu,* 19.9-fold enriched in vGluT2 vs vGAT), neuropeptide FF (*Npff,* 43.0-fold enriched in vGluT2 vs vGAT), neurotensin (*Nts,* 18.0-fold enriched in vGluT2 vs vGAT), tachykinin 2 (*Tac2*, encoding PPTB, the precursor of neurokinin B, 18.2-fold enriched in vGluT2 vs vGAT), thyrotropin releasing hormone (*Trh,* 7.4-fold enriched in vGluT2 vs vGAT) and urocortin 3 (*Ucn3,* 930 fold enriched in vGluT2 vs vGAT), adenylate cyclase activating polypeptide (*Adcyap1,* 46-fold enriched in vGluT2 vs vGAT) and corticotropin-releasing hormone (*Crh,* 66-fold enriched in vGluT2 vs vGAT). The comparison of vGluT2 with the vGAT neurons population yielded the same 12 genes, plus in addition tachykinin 1 (*Tac1*, encoding PPTA, the precursor of substance P and neurokinin A, sevenfold enriched in vGluT2 vs vGAT), which was the 56-most enriched gene in the vGluT2–Gad67 comparison. The strong enrichment and low read counts in vGAT and Gad67 samples (Tables S1, S2, S4) suggest that these neuropeptides are either exclusively expressed in excitatory interneurons or are present in only very few inhibitory interneurons. A few neuropeptides (Pnoc, *NPY* and *Pdyn*) were found enriched in inhibitory neurons. However, with only three out of the 50 most enriched genes, neuropeptide enrichment was far less common in inhibitory neurons than in excitatory populations. In order to more deeply investigate the expression patterns of the neuropeptide-encoding genes enriched in excitatory neurons, we employed fluorescent in situ hybridization (RNAscope). Of the 13 identified genes, two genes, adenylate cyclase activating polypeptide (*Adcyap1*) and corticotropin-releasing hormone (*Crh*), could not be detected reliably with in situ hybridization experiments. For the remaining 11 genes, we performed multiplex RNAscope experiments, combining up to three different neuropeptides at a time. Figure 5 (Fig. 5A–O) displays five of these combinations, in which every neuropeptide is represented at least once. Quantification of the co-expression of the depicted neuropeptides (Fig. 5C,F,I,L,O) suggested that most are expressed in different neuronal subtypes, yet one combination (*Tac2* and *Nmu*) displayed a high degree of co-expression (Fig. 5F). To systematically determine the overlap between the different neuropeptide-expressing populations, we quantified for each pair of neuropeptides the percentage of co-expressing neurons. As some neuropeptides displayed a wide range of expression levels, we performed separate analyses either including low expressors (threshold near to background levels) or taking only cells into account with an expression clearly above background (threshold > 20 dots per cell). We created two expression matrices summarizing the percent of co-expressing neurons either taking only medium to high expression levels into account (Fig. 6) or all neuropeptide expressing neurons irrespective of the expression level (Fig. S4). Restricting the analysis to the medium to high expressors led to more pronounced segregations, accentuating the absence or presence of co-expression. When focusing on medium to high-level expression, our analyses suggested that at least eight excitatory subpopulations are marked by either a single or a combination of several neuropeptides: 1. *Npff*^+^, 2. *Grp*^+^, 3. *Tac1*^+^, 4. *Tac2*^+^;*Nmu*^+^, 5. *Tac2*^+^;*Nmu*^−^; 6. *Nts*^+^, 7. *Cck*^+^,*Trh*^−^,*Ucn3*^−^, 8. *Cck*^+^;*Ucn3*^+^;*Trh*^+^. *Trh* and *Ucn3* display a high degree of overlap with each other (58% and 72%, respectively) and also highly overlap with *Cck* (86% and 91%, respectively). Conversely, only a minority of *Cck* neurons are *Trh* or *Ucn3* positive, suggesting that the *Trh* and *Ucn3* expressing neurons are a subpopulation of the *Cck* expressing neurons. Similarly, *Nmu* expressing neurons appear to define a subset of *Tac2* positive neurons (76% of the *Nmu* neurons are also *Tac2* positive and 25% of the *Tac* neurons are *Nmu* positive).

**Figure 7:**
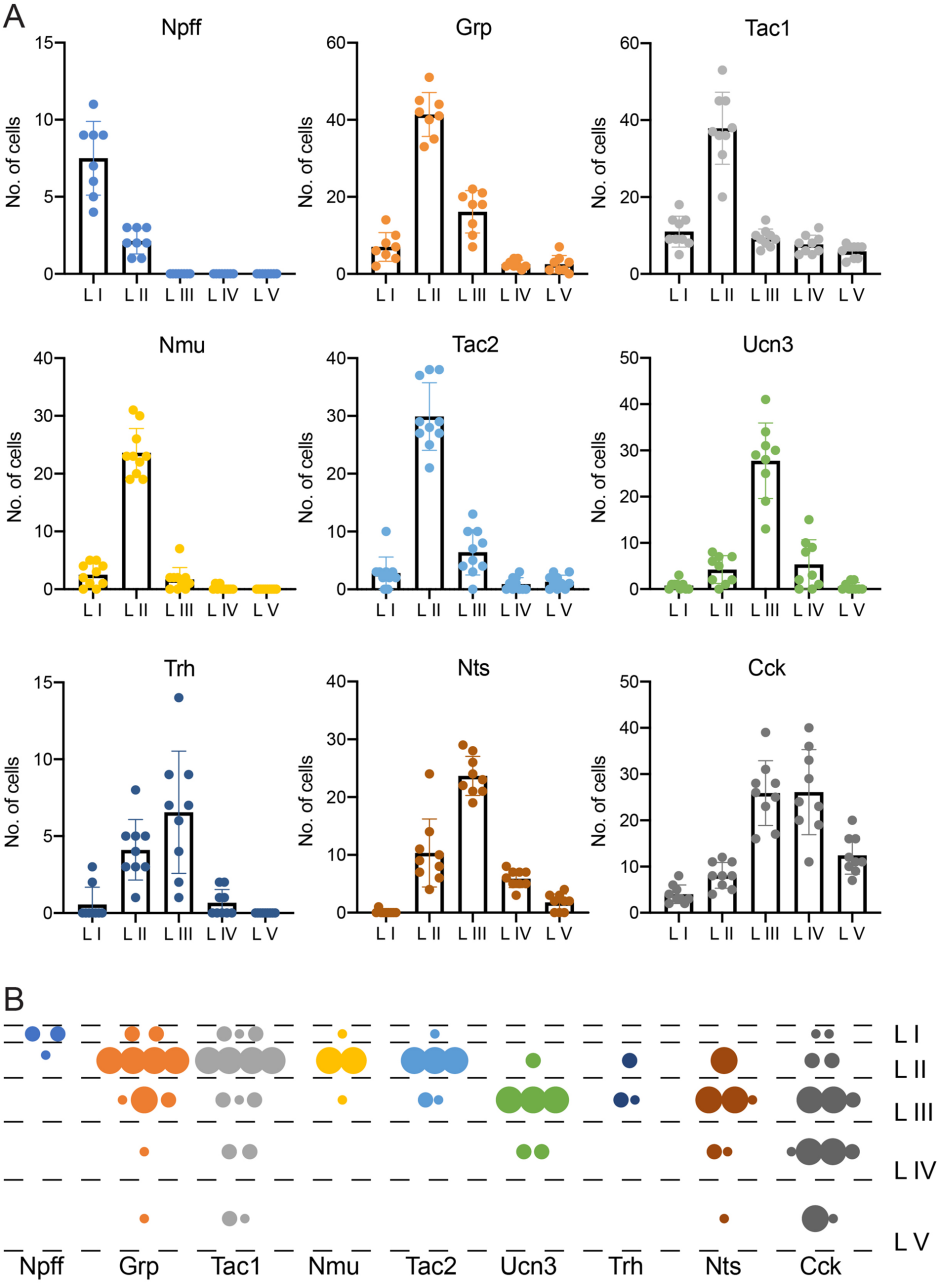
Laminar distribution of neuropeptides. (**A**) Number of cells per hemisection that express the depicted neuropeptide within the respective lamina of the spinal dorsal horn. Individual dots represent the number in a single hemisection (n = 8–10 hemisections from n = 3 mice). (**B**) Distribution of cells expressing the neuropeptide genes *Npff*, *Grp*, *Tac1*, *Nmu*, *Tac2*, *Ucn3*, *Trh*, *Nts* and *Cck* among the laminae of the dorsal spinal cord. Small dots = 2 cells, medium sized dots = 4 cells, large dots = 10 cells.

The expression patterns of several neuropeptides described above suggest a spatially restricted expression limited to only one or two spinal laminae (Fig. 5). To address the spatial distribution of each neuropeptide in more detail we imaged spinal cords at high resolution at the L3 and L4 levels. High resolution tiles were stitched, and the resulting images where aligned to reference sections from the spinal cord^26^ (Fig. S5). We then determined the number of cells expressing the respective neuropeptide in each lamina. Nine out of the eleven neuropeptides were predominantly expressed in only one or two spinal laminae (Fig. 7A). Npff neurons were concentrated in lamina I, Grp, Nmu, Tac1 and Tac2 neurons were present mainly in lamina II, whereas Nts, Trh and Ucn3 neurons predominated in lamina III. *Cck* neurons were mainly located in the deep dorsal horn (laminae III–V). In case of the remaining two neuropeptides, Nmb or Nms, expression patterns were weak and dispersed over most of the dorsal horn and were therefore not quantified. These data indicate that the different laminae of the spinal dorsal horn are patterned by the expression of unique combinations of only partially overlapping neuropeptide genes (Figs. 6, 7B).

## Discussion

In this study we have characterized the mRNA translation profiles (translatomes) of inhibitory (GABA/glycinergic) and excitatory (glutamatergic) spinal cord neurons. With this data we provide the first searchable dataset of neuron specific translatomes for the spinal cord. Pathway and Gene Ontology analyses of the translatomes of these two populations revealed that subtypes of neurotransmitter receptors and of down-stream signalling cascades differ between excitatory and inhibitory neurons and that the heterogeneity within the large population of excitatory dorsal horn neurons and their lamina organization is impressively mirrored by the expression of 11 neuropeptides or neuropeptide precursors.

### Correlation of transcriptome and translatome in spinal neurons

mRNA translation profiles obtained by the TRAP approach provide information about cell type-specific gene expression but without the cellular resolution obtained with single cell transcriptome profiling. However, the fraction of polysome-bound mRNA should more accurately reflect protein expression than approaches based on total or especially nuclear RNA analyses^18^. This is also supported by our study. The great majority of differentially expressed genes, revealed in our translatome comparisons, have previously been identified as marker genes of neuron subpopulations in studies using single cell RNAseq. However, we also found genes enriched in inhibitory and excitatory neurons that were either not detected with single cell RNAseq or detected only at levels that were too low to reliably assign them to a subpopulation (e.g. *Phox2a*). Other genes may have been filtered out to reduce artefacts (e.g. cell stress responses) potentially introduced by the necessary dissociation of the tissue (e.g. *Ighg3*). Hence, the sensitivity of the TRAP approach appears to be at least equal to single cell RNA sequencing. At the same time, our comparison of the most enriched genes versus data from single cell RNA sequencing also suggests a relatively high congruence between the transcriptome and the translatome. A comprehensive comparison of our translatome data with a recently developed harmonized transcriptional atlas of the spinal cord^27^ may provide additional insights into the degree to which transcribed mRNAs are also translated.

### Differential use of neurotransmitter and neuromodulator signaling pathways

Spinal inhibitory and excitatory neurons receive synaptic inputs from a wide variety of peripheral, spinal and supraspinal neurons, including glutamatergic, GABAergic, glycinergic, serotonergic, cholinergic, and adrenergic neurons. We found that both inhibitory and excitatory spinal neurons express receptors for all these neurotransmitters (Tables S6–15). However, in some cases, neurotransmitter signaling appears to occur via different receptor subtypes and accordingly down-stream signaling cascades. Expression of several glutamate receptors of the kainate family (Grik1-3) is enriched in inhibitory spinal neurons. Kainate receptors display different modes of signaling and a metabotropic action of kainate receptors has been suggested to be involved in presynaptic regulation and inhibition of GABA release^28,29^. A preferential expression of kainate receptors on inhibitory spinal neurons might therefore be involved in a glutamate-mediated inhibition of GABA (and potentially glycine) release. Even more striking is the enrichment of several serotonin receptors on inhibitory neurons with the most pronounced example being Htr3a, one of the two ionotropic serotonin receptor subtypes. Expression of Htr3a is more than eightfold enriched in inhibitory neurons and in situ hybridization indicated expression restricted to the deep dorsal horn. It is therefore conceivable that serotonin released in the spinal cord from descending supraspinal neurons has a differential effect on excitatory and inhibitory neurons.

### Lamina specific expression of neuropeptides suggests a neuropeptide code involved in modality specific sensory processing

Our data confirm and extend previous single cell studies that identified neuropeptides as markers of excitatory neuronal subtypes^9,10^. Our multiplex in situ hybridization experiments demonstrate a lamina-specific distribution of many of the enriched neuropeptides with rather limited co-expression between different neuropeptides. In total, we find that the 11 different neuropeptides analysed in this study span dorsal horn laminae I–V, thus potentially setting up a map for a neuropeptide sensory signalling code. This is in good agreement with recent literature, as a number of these genes have either already been suggested to define specific subsets of spinal neurons^11,30–32^ or have already been used as driver genes for functional manipulation of spinal neuron subtypes^14,33–39^. These studies demonstrate that neuronal subpopulations marked by the expression of specific neuropeptides exert modality specific functions in sensory processing. Grp+ and Ucn3+ neurons are required for the relay of pruriceptive information^33,37,39^, Tac1+ neurons are required for pain associated coping behaviours, Nts+ neurons are suggested to contribute to temporal summation of nociceptive input and “wide-up”^34^ and *Cck* expressing neurons are crucially involved in the development of mechanical allodynia^35,38^. More importantly, emerging evidence suggests that neuropeptides do not only label specific subtypes of spinal neurons but are themselves crucial for modality specific sensory processing. The release of the neuropeptides Cck and Nts has been associated with pronociceptive or antinociceptive effects^40–42^. Signalling via the gastrin releasing peptide (Grp), which is concentrated in lamina II, is critical for itch perception in mice^33,39,43,44^ and required for successful signal transmission from second order Grp to third order Grp receptor (GRPR) positive neurons in lamina II of the spinal cord^45^. Another example of a neuropeptide modulating pruriceptive information at the spinal level is somatostatin (SST). We found SST to be enriched in spinal excitatory neurons but we also detected substantial expression in inhibitory neurons (Table S1). SST expression in inhibitory dorsal horn neurons has been previously detected by Proudlock et al.^46^ and Haring et al.^9^ (see http://linnarssonlab.org/dorsalhorn/) but was not reported by others^47,48^. However, SST is primarily expressed in peripheral neurons and in excitatory lamina II dorsal horn neurons. After release of SST from peripheral sensory neurons or spinal dorsal horn neurons it inhibits inhibitory dynorphin (Dyn) neurons, which in turn inhibit Grp receptor expressing neurons^49^. SST release thereby facilitates pruriceptive signalling. In this context, it is interesting to note that while the majority of spinal SST expressing neurons are excitatory (glutamatergic), the effect of SST on Dyn neurons is inhibitory. These studies demonstrate that neuropeptides are able to add an additional layer of complexity to neuronal communication and may in certain cases dominate over the effect of fast-acting neurotransmitters such as glutamate, GABA and glycine. The lamina specific expression of many neuropeptides in dorsal horn neurons suggests that distinct groups of neuropeptide expressing cells are activated by a restricted subset of primary afferents. Hence, distinct neuropeptides might only be released in response to certain sensory stimuli.

## Conclusions

The present study has utilized three newly generated lines of BAC TRAP transgenic mice that allow cell type-specific translatome analyses of excitatory glutamatergic and inhibitory GABAergic/glycinergic neurons. In the present study, these mice allowed us to identify several signalling pathways enriched in distinct neuron populations of the dorsal horn. Particularly interesting to us appeared an enrichment of neuropeptide signalling in different populations of excitatory neurons with several neuropeptides exhibiting a rather lamina specific distribution suggesting an important role in modality specific sensory processing. While such a specific involvement is already known for some of these neuropeptides, the function of others is still obscure. The mouse lines described in this report and the accompanying translatome database should hence be well suited to reveal hitherto unknown mechanisms of normal sensory processing and their changes in disease states.

## Materials and methods

### Animals

Experiments were performed on 6–12-week-old mice kept at a 12:12 h light/dark cycle that received food and water ad libitum and were kept in a 12 h light/dark cycle. All methods were carried out in accordance with relevant guidelines and regulations. All animal experiments have been applied for. They have been evaluated by the Commission on Animal Experimentation of the canton of Zurich. The Commission recommended the cantonal veterinary office (Kanton Zürich, Gesundheitsdirektion, Veterinäramt, Zollstrasse 20, CH-8090 Zürich) to accept the respective applications. Subsequently, the cantonal veterinary office granted the following licenses; license ZH063/2010, ZH074/2013, ZH031/2016 and ZH011/2019. These licenses cover all experiments described in this study. Animal experiments were conducted adhering to the ARRIVE guidelines^50^.

### Generation of vGluT2::bacTRAP, vGAT::bacTRAP and Gad67::bacTRAP mouse lines

The vGluT2::bacTRAP mouse line was generated by placing cDNA for the eGFP-L10a (TRAP) transgene, followed by the bovine growth hormone polyadenylation (poly(A)) signal in frame into exon 2 of the *vGluT2* (*Slc17a6*) gene (on BAC clone RPE23-84M15), using homologous recombination in bacteria^51^, replacing most of exon 2 of the *vGluT2* gene. The start codon (ATG) in exon 1 was mutated to an XhoI restriction site. For the vGAT::bacTRAP mouse line the transgene was placed in frame into the end of exon 1 of the *vGAT* (*Slc32a1*) gene, replacing it partially (on BAC clone RPE23-392P11). For the Gad67::bacTRAP mouse line the transgene was placed in frame into the end of exon 2 of the *Gad67* (*Gad1*) gene, replacing it partially (on BAC clone RP24-256C12). BAC DNA was injected into C57BL/6 oocytes. The thereby generated mice were screened for the presence of the eGFP-L10a transgene and for the two BAC ends by PCR. The transgenic mouse lines were maintained on a C57BL/6J (Black 6, The Jackson Laboratory, Stock No: 000664) background. C57BL/6J mice were also used as wild type controls. Mouse breeding and maintenance were provided by the Laboratory Animal Services Center (LASC) of the University of Zurich.

### Genotyping PCRs

The presence of the eGFP-L10a transgene in the three different mouse lines was determined using a specific PCR for the transgene or a generic PCR against eGFP with one of the following primer pairs:

**Table:** 

	Forward primer	Reverse primer	Product length	Annealing temp
vGuT2::bacTRAP	GGAAGTGCCTAACCACAGGCGT	CTGGTTCCCGTGCAGGACT	873 bp	56–70 °C
vGAT::bacTRAP	CGAGGGTCATGAGCCAGAGC	CTGGTTCCCGTGCAGGACT	950 bp	68 °C
Gad67::bacTRAP	CTCTCCCTTCTGTTTGCAGCCT	CTGGTTCCCGTGCAGGACT	904 bp	66 °C
eGFP 1	CTATATCATGGCCGACAAGC	ACTGGGTGCTCAGGTAGTGG	164 bp	56 °C
eGFP 2	GGGCGAGGAGCTGTTCACC	ACTTGTACAGCTCGTCCATGCC	700 bp	65 °C

### Immunohistochemistry (IHC)

For mouse line validation, adult (> 2 months old), male and female bacTRAP were used. Mice were transcardially perfused with approximately 20 mL of ice-cold artificial cerebrospinal fluid (ACSF, pH 7.4) or phosphate buffered saline (PBS, pH 7.4), followed by 100 mL of 2 or 4% ice-cold paraformaldehyde (PFA, in PBS or 0.1 M Sodium phosphate buffer (PB), pH 7.4). The lumbar spinal cord was dissected and post-fixed for 1.5–2 h in 4% PFA solution (in PBS or 0.1 M PB, pH 7.4), followed by incubation in 30% sucrose (in PBS or 0.1 M PB, pH 7.4) for cryoprotection at 4 °C overnight. Cryoprotected spinal cords were embedded in NEG50 frozen section medium (Richard-Allen Scientific) and stored at − 80 °C until cutting into 25 μm thick sections on a Hyrax C60 Cryostat (Carl Zeiss). Sections were mounted on Superfrost Plus glass slides (Thermo Scientific, Zurich, Switzerland) and stored at − 80 °C.

For immunofluorescence staining the slides were washed for 5 min in PBS to remove the embedding medium, followed by blocking with 5% normal donkey serum (NDS, AbD Serotec, RRID:SCR_008898) in 0.1% Triton X-100-PBS for at least 30 min at room temperature (RT). Sections were incubated with primary antibodies (see resource table) in the blocking solution at 4 °C overnight, followed by three washes in PBS for 5 min and incubation with secondary antibodies (fluorophore-coupled donkey antibodies, Jackson ImmunoResearch) in blocking solution at RT for 30–60 min. Subsequently, sections were washed in PBS and incubated with 4′,6-diamidino-2-phenylindole (DAPI, 500 ng/ml in PBS) for 10 min and again washed in PBS. Finally, sections were covered with DAKO fluorescent mounting medium (Dako, RRID:SCR_013530) and coverslips.

### In situ hybridization (ISH)

For ISHs, 6 weeks old, naïve male C57BL/6J mice were used. For spinal cord preparation, the vertebral column containing the lumbar part of the spinal cord were dissected, immediately after decapitation of the mouse. A syringe filled with ice-cold, RNAse-free PBS was fitted tightly against the caudal opening of the spinal canal and pressure was applied to press the spinal cord out, which was timed to the lumbar part. After dissection, spinal cords were snap frozen in liquid nitrogen and stored at − 80 °C until embedding in NEG50 frozen section medium (Richard-Allen Scientific). Frozen blocks were again stored at − 80 °C until cutting into 16–25 μm thick sections and mounted as described for IHC.

For single-plex chromogenic ISH the manual RNAscope 2.5 HD BROWN Assay (ACD, Bio-Techne, catalog no. 322300) was used. The manufacturer’s pretreatment protocol for fresh frozen tissue (document no. 320536-TN, rev. date 11112016) and detection protocol (document no. 322310-USM, rev. date 11052015) were followed. Hybridization with Amp 5 was increased from 30 to 60 min for all probes in order to increase the signal. Counterstaining in 50% hematoxylin was reduced from 2 min to 30 s. Probes are listed in the resource table.

For multiplex fluorescent ISH (FISH) the manual RNAscope Multiplex Fluorescent Assay (ACD, Bio-Techne, catalog no. 320850) was used. The manufacturer’s pretreatment protocol for fresh frozen tissue (document no. 320513, rev. date 11052015) and detection protocol (document no. 320293-UM, rev. date 03142017) were followed. The fluorophore alternatives (Amp 4 Alt) were chosen in such a way, that when possible the weakest expressing gene would lie in red channel (Atto 550) and not in the far-red channel (Atto 647). The 3-plex negative control probe was amplified with the corresponding Amp 4 Alt. Probes are listed in the resource table. For every neuropeptide combination, one slide with four lumbar spinal cord sections of four animals, each, was reacted.

### Image acquisition and analysis

Bright field imaging of ISHs and fluorescent imaging of FISHs and IHCs was done using the following microscopes:

**Table:** 

Microscope	Objectives	Software	Purpose
Zeiss Axio Scan.Z1 slidescanner	5×/0.25 Fluar air 10×/0.45 Plan-Apochromat air	ZEN 2 slidescan (blue edition)	Bright field images of chromogenic ISHs
Zeiss LSM710 confocal microscope	20×/0.8 Plan‐Apochromat air 40×/1.3 EC Plan‐Neofluar oil	ZEN 2011 (black edition)	Fluorescent images for mouse line validation
Zeiss LSM700 confocal microscope	25×/0.8 Plan‐Neofluar oil 40x/1.4 LCI Plan-Apochromat oil	Zen 2011 (black edition)	Overview images of multiplex FISH
Zeiss LSM 800 with Airyscan/confocal	25×/0.8 Plan‐Neofluar oil 40×/1.4 Plan-Apochromat oil	ZEN 2.3 (blue edition)	Overview and analysis images of multiplex FISH

For fluorescent imaging, the pinhole was set to 1 airy unit for every channel, which were scanned sequentially to avoid overlapping emission spectra or with a combination of the ultraviolet and infrared channel in one track, where emission spectra overlap is minimal.

For the validation of the correct expression of the eGFP-L10a transgene, the 40× objectives on the LSM 700 or 710 were used with a zoom of 0.6× to acquire a tile scan over the complete lumbar dorsal horn (Pixel size: × 0.346 μm, y 0.346 μm, z 1.628/2.487 μm). A small z-stack (usually, 3.3 μm within 3 optical sections) at a bit depth of 8 was acquired. For the analysis, the cell counter plugin of ImageJ was used. Manual counting was done in the middle optical section; the other sections were used as an aid to distinguish between neighboring cells. Three hemi-sections from three animals of each mouse line were analyzed. Ratios were calculated per animal and then averaged.

For the quantification of the co-expression of the neuropeptides with multiplex FISH, the 40× objective on the LSM 800 was used with a zoom of 2× and an image size of 1024 × 1024 px (pixel size: × 0.078 μm, y 0.078 μm). An array of non-overlapping images that covers the complete dorsal horn was scanned at a bit depth of 16. Analysis was done with CellProfiler 2.2.0 software^52^. The cell profiler pipeline can be provided upon request. Briefly, nuclei were detected using the *IdentifyPrimaryObjects* module and expanded with the *IdentifySecondaryObjects* module to define the cell soma (from now on referred to as “cell”). The signal dots were detected using the *IdentifyPrimaryObjects* module, adapting the “threshold correction factor” and the “lower and upper bounds on threshold” for every slide (neuropeptide combination) to minimize false positive and false negative detection. The signal dots of the different channels were assigned to the cells they lie in using the *RelateObjects* module. The number of cells and related signal dots (per channel) were exported using the *ExportToSpreadsheet* module. Images with artefacts or high background were manually excluded from the automatic analysis in order to avoid false positive signal. Per slide (neuropeptide combination), three animals with two hemi-sections per animal were analyzed. Additionally, per combination one hemi-section hybridized with the 3-plex negative control probe and amplified with the same Amp 4 Alt was imaged with the same microscope setting and analyzed with the same CellProfiler setting in order to determine the background.

Data analysis was conducted with R. To determine the signal background, the 3-plex negative control was analyzed. For every combination and channel, the 90th percentile of dots per cell in the negative control was determined. This value was set as the threshold above which a cell is counted as positive for the respective signal. 20 dots per cell were set as a threshold for high expression. The number of single, double and triple-positive cells was counted. Ratios were calculated per animal and then averaged.

### Cell-type-specific polysomal mRNA isolation from excitatory and inhibitory spinal cord neurons

To specifically isolate polysomal mRNA from genetically targeted cells, the three bacTRAP mouse lines described above were used and the protocol published by Heiman, et al.^53^ was followed. In brief, solutions and the affinity matrix were prepared as described. For the affinity matrix of Gad67::bacTRAP 300 μL and of vGluT2::bacTRAP and vGAT::bacTRAP 150 μL Streptavidin MyOne T1 Dynabeads and the corresponding volumes of biotinylated protein L and GFP antibodies 19C8 and 19F7 were used in a final volume of 200 μL.

Per mouse line three male mice per mouse line, aged between 8 and 10 weeks were used. The vertebral column containing the lumbar part of the spinal cord was dissected, immediately after decapitation. A syringe filled with ice-cold dissection buffer was fitted tightly against the caudal opening of the spinal canal and pressure was applied to press the spinal cord out, which was trimmed to the lumbar part. For the neuropathic pain analysis, the lumbar spinal cord was bisected along the midline and the left side (ipsilateral to the peripheral nerve surgery) was further processed. The tissue was washed, homogenized, centrifuged, lysed and again centrifuged as described in the protocol by Heiman et al.^53^.

Immunopurification was done as described by Heiman et al.^53^. RNA purification was done using the RNeasy Plus Micro Kit (Qiagen, catalog no. 74034). RNA was eluted from beads with 350 μl Buffer RLT Plus from the kit, supplemented with 40 μM dithiothreitol (DTT, Thermo Fisher Scientific, catalog no. R0861) and then processed according to the manufacturer’s protocol. RNA concentration was determined using the RiboGreen fluorescence assay (Thermo Fisher Scientific, catalog no. R11491) on a NanoDrop 3300 microvolume fluorospectrometer. RNA quality was assessed by high sensitivity RNA screen tape on an Agilent 2200 TapeStation. RNA samples with RINe values of 6.8–7.8 were used for sequencing library construction.

### Sequencing library construction, next-generation sequencing (NGS), mapping and differential gene expression analysis (DGEA)

The sequencing library was prepared using the Ovation Mouse RNA-Seq System 1–16 kit (NuGEN, catalog no. 0348-32). The manufacturer’s protocol was followed. 13–20 ng RNA were used as input material for the inhibitory—excitatory comparison and 30 ng RNA for the neuropathic pain analysis. The optional cDNA fragmentation step was performed on a Covaris S220 focused-ultrasonicator (Covaris) to obtain a median fragment size of approx. 300 bp. As recommended, the optimal cycle number for amplification was determined by qPCR (cycle number within the exponential phase of the amplification plot) and turned out to be 18.

Sequencing of the library, mapping to the reference genome, as well as DGEA were performed by the Functional Genomics Center Zurich (FGCZ). NGS was done on two lanes of a HiSeq 2500 system (illumina) with single-end sequencing of 125 bp. Mapping was done in R using the STAR package, version 2.4.2a [68]. The reference genome build Mus_musculus Ensembl GRCm38 release 80 was used for alignment. Reads mapped to exons were counted using countOverlaps in GenomicRanges version 1.20.5 (parameters: countNonredundant = TRUE, minFeatureOverlap = 10, minMapQuality = 10, keepMultiHits = TRUE)^54^. DGEA was done in R using either the package edgeR 3.10.2 or DESeq2 1.8.1 [69–71]. Bioinformatic analysis was performed using the R package ezRun (https://github.com/uzh/ezRun) within the data analysis framework SUSHI^55^. Quality checkpoints^56^, such as quality control of the alignment and count results, were implemented in ezRun (https://github.com/uzh/ezRun) and applied throughout the analysis workflow to ensure correct data interpretation. In details, read alignments were comprehensively evaluated using the RnaBamStatsApp (https://github.com/uzh/ezRun/blob/master/R/app-RnaBamStats.R) in the ezRun package, in terms of different aspects of RNA-seq experiments, such as sequence quality, gDNA and rRNA contamination, GC/PCR/sequence bias, sequencing depth, strand specificity, coverage uniformity and read distribution over the genome annotation. Gene counts were comprehensively evaluated using the CountQCApp (https://github.com/uzh/ezRun/blob/master/R/app-countQC.R) in the same R package, including sample correlation, sample clustering, as well as clustering of high variance features.

### Gene Ontology (GO) term and pathway enrichment analyses

GO term enrichment analysis was done using the online PANTHER tool (http://pantherdb.org/). Genes significantly (FDR ≤ 0.05) and ≥ 2-fold enriched in excitatory (vGluT2) over inhibitory (vGAT or Gad67) and vice versa were analyzed. In the PANTHER overrepresentation test, the sets of enriched genes were compared to the set of all expressed genes as a reference. GO molecular function (MF) complete was used as the annotation data set. The significantly overrepresented (FDR ≤ 0.05) MFs were analyzed. When several GO terms of a hierarchy were significantly overrepresented, only the most specific GO term was displayed in the graphical representation. For the analysis of Gad67 vs. vGluT2, two redundant GO terms were not displayed in the graphical representation in order to allow comprehensibility.

Pathway analysis was done with Ingenuity Pathway Analysis (IPA, QIAGEN). Genes significantly (FDR ≤ 0.05) and ≥ 2-fold enriched in excitatory (vGluT2) over inhibitory (vGAT or Gad67) and vice versa were analyzed. To get a coarse classification of the enriched genes into subcellular location and function (“type”), these parameters were exported from the software.

### Resources

**Table:** 

Reagent	Resource	Identifier
**Antibodies (dilution)**		
goat anti-Pax2 (1:200–1:400)	R & D Systems (Minneapolis, MN, USA)	RRID:AB_10889828
guinea pig anti-Lmx1b (1:10,000)	Dr. Carmen Birchmeier	^57^
rabbit anti-GFP (1:2000)	Molecular Probes	RRID:AB_221570
mouse anti-NeuN (1:1000)	Millipore	RRID:AB_2298772
guinea pig anti-NeuN (1:1000)	SynapticSystems	RRID:AB_2619988
**RNAscope multiplex FISH probes**		
Mm-Cck	ACD, Bio-Techne	402271
Mm-Grp	ACD, Bio-Techne	317861
Mm-Grp-C2	ACD, Bio-Techne	317861-C2
Mm-Nmb	ACD, Bio-Techne	459931
Mm-Nmb-C3	ACD, Bio-Techne	459931-C3
Mm-Nms	ACD, Bio-Techne	472331
Mm-Nms-C3	ACD, Bio-Techne	472331-C3
Mm-Nmu	ACD, Bio-Techne	446831
Mm-Npff	ACD, Bio-Techne	479901
Mm-Nts-C2	ACD, Bio-Techne	420441-C2
Mm-Tac1-C3	ACD, Bio-Techne	410351-C3
Mm-Tac2	ACD, Bio-Techne	446391
Mm-Tac2-C2	ACD, Bio-Techne	446391-C2
Mm-Trh	ACD, Bio-Techne	436811
Mm-Ucn3	ACD, Bio-Techne	464861
Mm-Barhl1	ACD, Bio-Techne	520561
Mm-Phox2a	ACD, Bio-Techne	520371
Mm-Ighg3	ACD, Bio-Techne	514611
Positive control probe Mm-Ppib	ACD, Bio-Techne	313911
Negative control probe-DapB	ACD, Bio-Techne	310043
3-plex negative control probe	ACD, Bio-Techne	320871

## Supplementary Information

Supplementary Information 1.

Supplementary Information 2.

Supplementary Information 3.
