# Supplementary material for: Neuron-specific spinal cord translatomes reveal a neuropeptide code for mouse dorsal horn excitatory neurons
Source: Sci Rep. 2021 Mar 4;11:5232. doi: 10.1038/s41598-021-84667-y (PMC7933427; doi:10.1038/s41598-021-84667-y)
Supplement: Supplementary file 1 — Supplementary Information 1. [file 41598_2021_84667_MOESM1_ESM.pdf]

Neuron-Specific Spinal Cord Translatomes Reveal a Neuropeptide Code for Mouse Dorsal Horn Excitatory Neurons

[1,2]Rebecca Rani Das Gupta, [1]Louis Scheurer, [3]Pawel Pelczar, [1*]Hendrik Wildner, [1,2*]Hanns Ulrich Zeilhofer
[1]Institute of Pharmacology and Toxicology, University of Zurich, Winterthurerstrasse 190, CH-8057 Zürich, Switzerland.
[2]Institute of Pharmaceutical Sciences, Swiss Federal Institute of Technology (ETH) Zurich, Vladimir-Prelog-Weg 1-5/10, CH-8090 Zurich, Switzerland
[3]Center for Transgenic Models, University of Basel, 4001 Basel, Switzerland.

*corresponding authors: Dr. H.U. Zeilhofer & Dr. H. Wildner, [1]Institute of Pharmacology and Toxicology, University of Zurich, Winterthurerstrasse 190, CH-8057 Zürich, Switzerland.
Phone: +41 44 63 55938
Fax: +41 44 635 59 88
e-mail: zeilhofer@pharma.uzh.ch
hwildner@pharma.uzh.ch

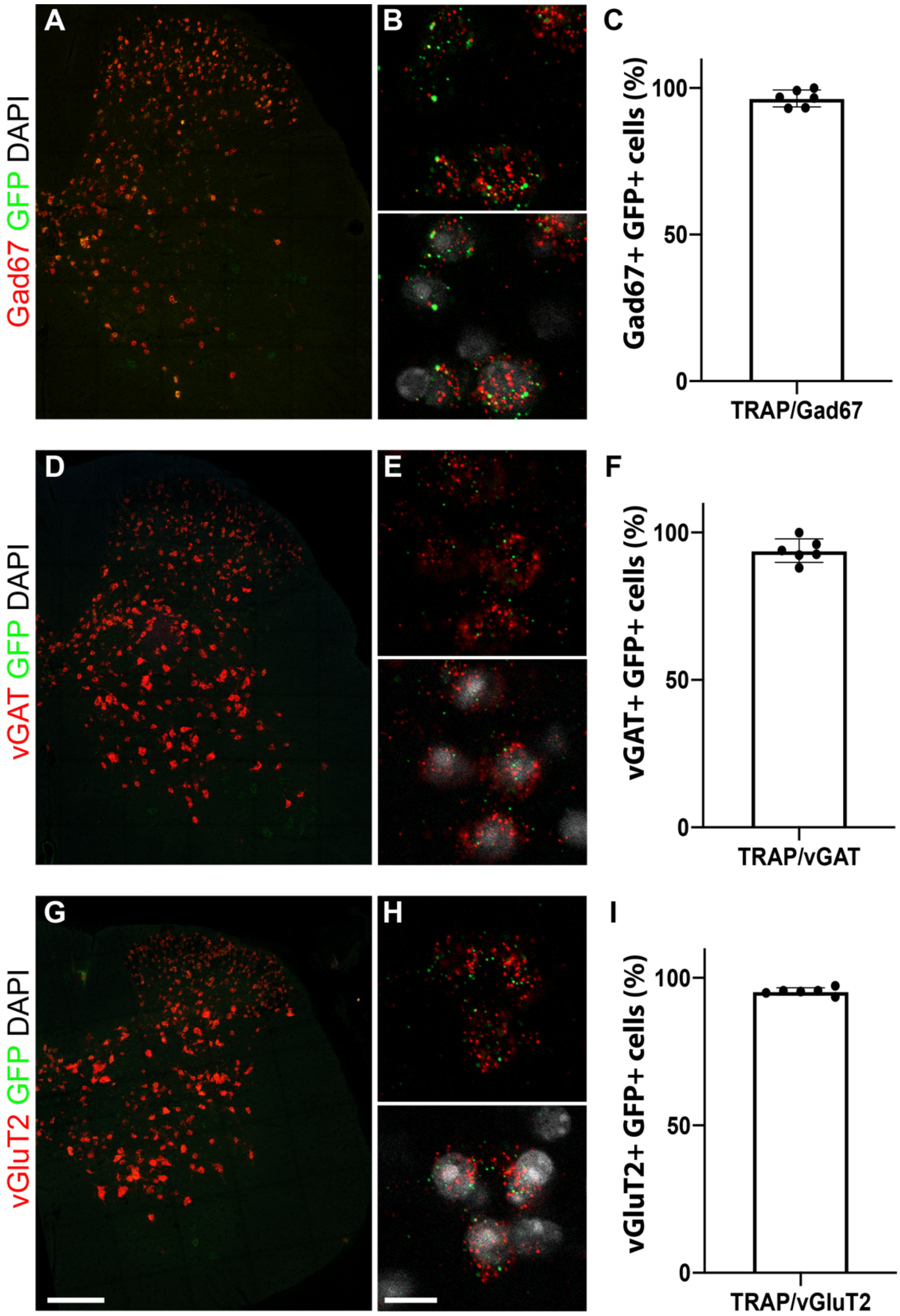

Figure S1 TRAP transgene expression in endogenous *Gad67*+, *vGAT*+ and *vGluT2*+ neurons. Multiplex *in situ* hybridization using RNAscope probes against *GFP* (A-H) depicted in green*, Gad67* (A, B), *vGAT* (D, E) or *vGluT2* (G, H) depicted in red*.* Note, L10a-eGFP expression is difficult to see in the low-resolution images (A, D and G) as expression from the respective transgenes appears to be lower than expression of from the corresponding endogenous loci. High resolution images (B, E and H) indicate a high level of co-epression of the respective endogenous gene and the TRAP transgene. (C, F, and G) quantification of the percentage of Gad67+, vGAT+ or vGluT2+ neurons that also express the TRAP transgene (GFP) in the respective bacTRAP transgenic mouse line. Scale bar: (A, D, G) 200 µm, (B, E and H) 10µm. The error bar represents the standard deviation.

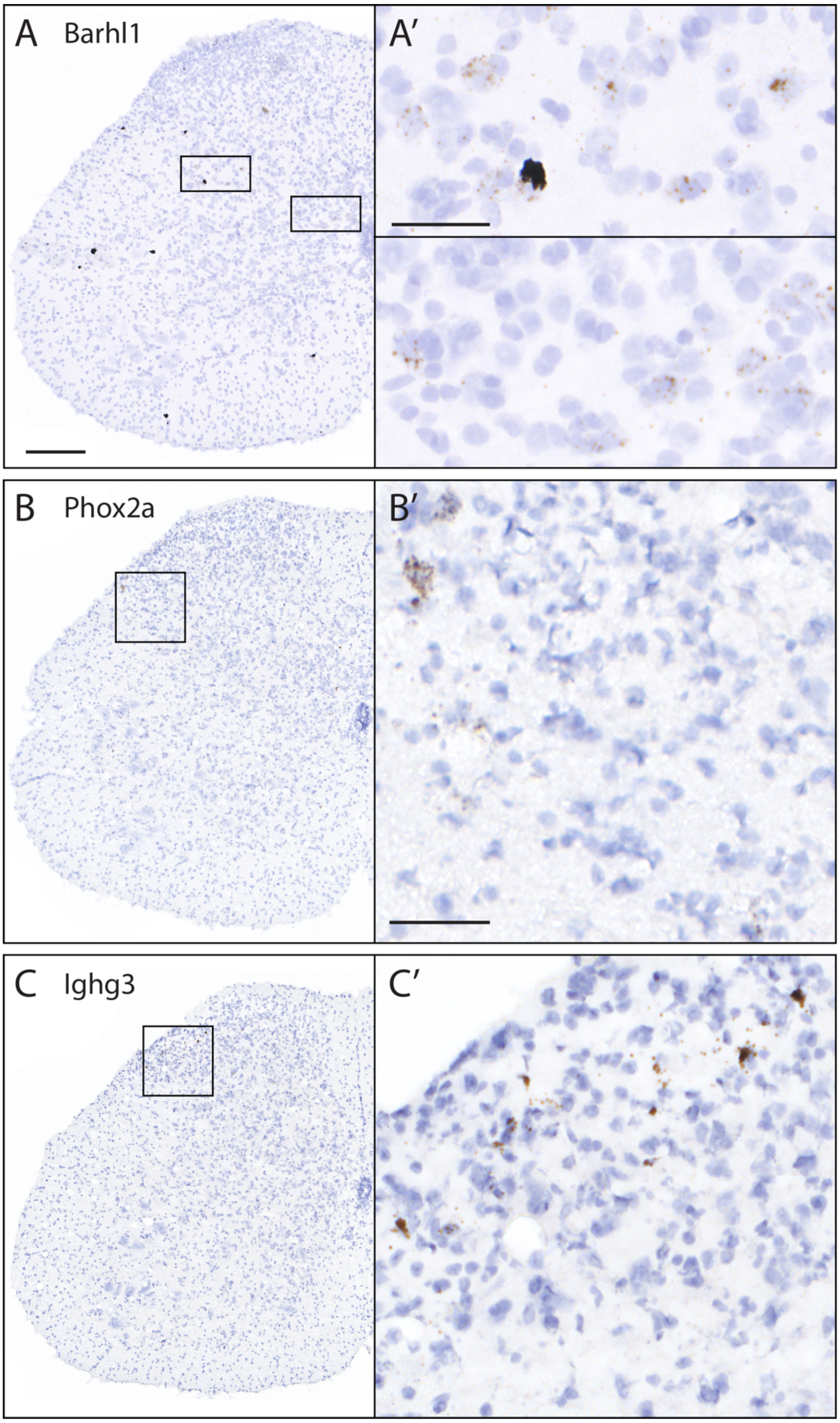

Figure S2 Confirmation of expression of genes with low detection levels, that were not detected in single-cell sequencing by Häring et al. [9].
In situ hybridization using RNAscope probes directed against (A) transcription factor BarH-like 1 (*Barhl1*), (B) transcription factor paired-like homeobox 2a (*Phox2a*), (C) Immunoglobulin heavy constant gamma 3 (*Ighg3*). Scale bar: (A, B, C) 200 µm, (A’, B’, C’) 50µm

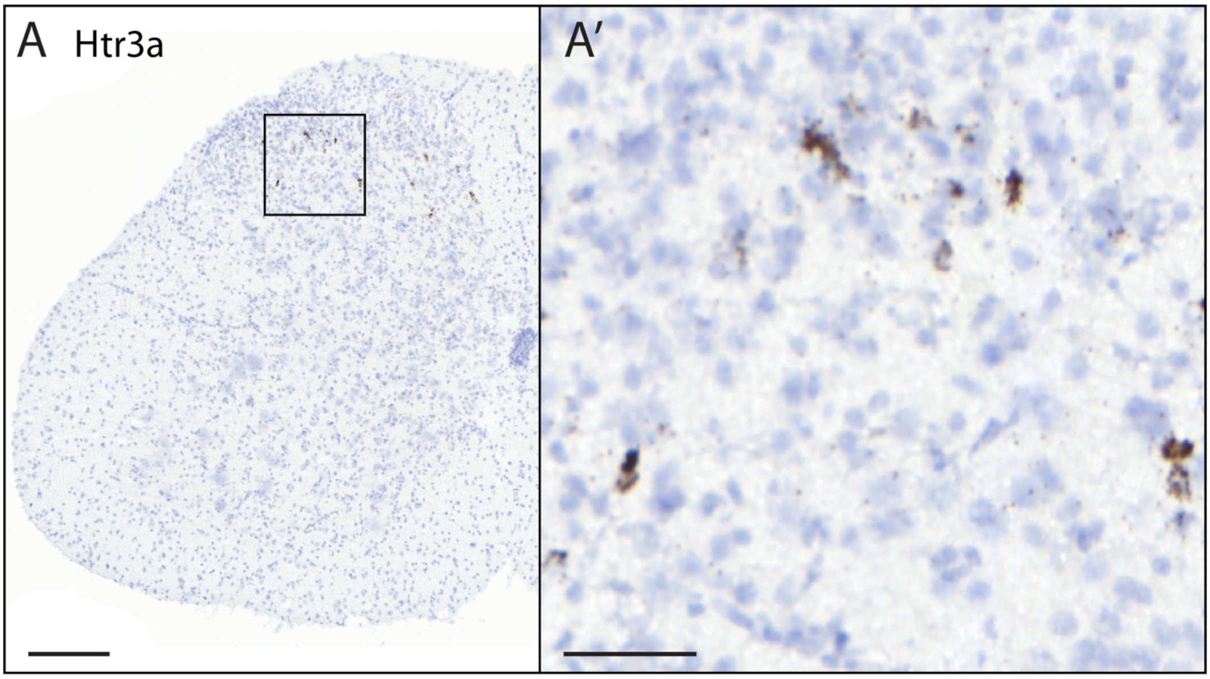

Figure S3 Expression pattern analysis of the ionotropic serotonin receptor Htr3a
Expression pattern analysis by *in situ* hybridization using a RNAscope probe directed against the mRNA encoding *Htr3a.*
Scale bar: (A) 200 µm, (A') 50 µm

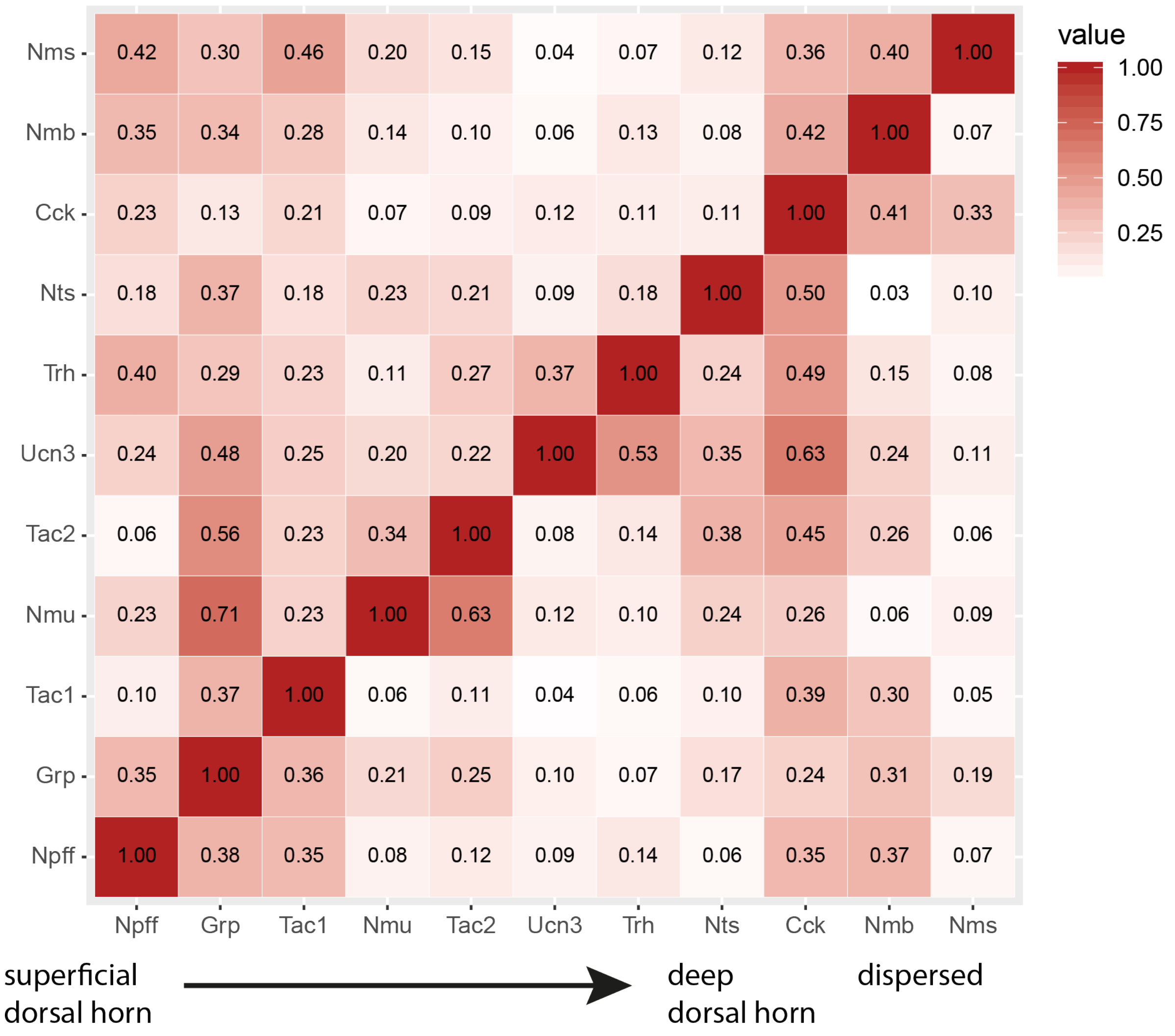

Figure S4 Co-expression matrix of eleven neuropeptides expressed in excitatory neurons (including low level expressors).

Co-expression within all cells (including all low expressors) expressing a neuropeptide. Fraction of cells expressing the neuropeptide on the left, that also express the neuropeptide at the bottom. For example, 42 % of all Nms-expressing cells, also express Npff. Color key indicates co-expression with light colors representing low co-expression and dark values representing high co-expression.

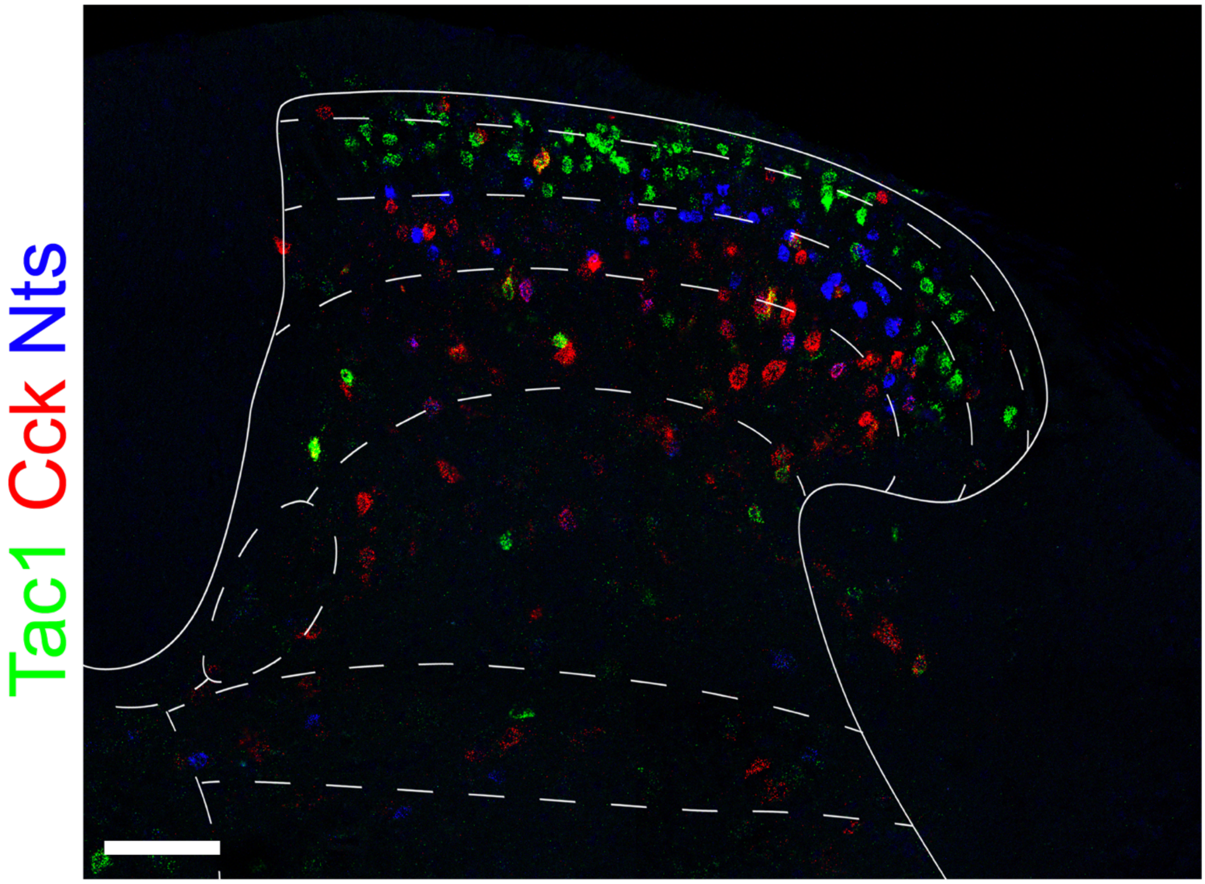

Figure S5 Representative example image indicating Rexed laminae in a dorsal spinal horn at spinal level L3 after multiplex fluorescent in situ hybridization. Spinal cord section has been reacted with probes directed against *Tac1, Cck* and *Nts.* Solid line outlines the spinal grey matter. Dotted lines indicate the borders of spinal laminae I-VI. Dotted oval outlines the dorsal nucleus. Scale bar: 100 µm
