# Supplementary material for: Neuron-specific spinal cord translatomes reveal a neuropeptide code for mouse dorsal horn excitatory neurons
Source: Sci Rep. 2021 Mar 4;11:5232. doi: 10.1038/s41598-021-84667-y (PMC7933427; doi:10.1038/s41598-021-84667-y)
Supplement: Supplementary file 3 — Supplementary Information 3. [file 41598_2021_84667_MOESM3_ESM.pdf]

Neuron-Specific Spinal Cord Translatomes Reveal a Neuropeptide Code for Mouse Dorsal Horn Excitatory Neurons

[1,2]Rebecca Rani Das Gupta, [1]Louis Scheurer, [3]Pawel Pelczar, [1*]Hendrik Wildner, [1,2*]Hanns Ulrich Zeilhofer

[1]Institute of Pharmacology and Toxicology, University of Zurich, Winterthurerstrasse 190, CH-8057 Zürich, Switzerland.

[2]Institute of Pharmaceutical Sciences, Swiss Federal Institute of Technology (ETH) Zurich, Vladimir-Prelog-Weg 1-5/10, CH-8090 Zurich, Switzerland

[3]Center for Transgenic Models, University of Basel, 4001 Basel, Switzerland.

*corresponding authors: Dr. H.U. Zeilhofer & Dr. H. Wildner, [1]Institute of Pharmacology and Toxicology, University of Zurich, Winterthurerstrasse 190, CH-8057 Zürich, Switzerland.

Phone: +41 44 63 55938

Fax: +41 44 635 59 88

e-mail: zeilhofer@pharma.uzh.ch

hwildner@pharma.uzh.ch

Enriched genes

		vGluT2 [norm. count]	vGAT [norm. count]	Gad67 [norm. count]	vGluT2 vs. vGAT		vGluT2 vs. Gad67		neuronal cell type
**Gene**	**Group**	**Mean ± Stddev**	**Mean ± Stddev**	**Mean ± Stddev**	**log2 Ratio**	**FDR**	**log2 Ratio**	**FDR**	**sc Data**
Ucn3	NP	106 ± 34	0 ± 0	5.9 ± 10.2	9.87	8.52E-14	4.18	4.60E-04	Glut3
Lhx2	TF	199 ± 35	0.96 ± 1.67	0 ± 0	7.62	9.29E-26	10.71	2.02E-35	nd
Olig3	TF	176 ± 28	1.56 ± 1.86	12.2 ± 7.1	6.58	5.80E-20	3.82	4.92E-13	nd
Barhl1	TF	382 ± 38	4.3 ± 2.9	0 ± 0	6.4	3.25E-49	11.65	5.24E-74	nd
Shox2	TF	765 ± 32	11.5 ± 10.4	25.6 ± 20.9	6.12	7.88E-35	4.91	9.81E-21	nd
Crh	NP	219 ± 44	3 ± 2.6	0 ± 0	6.05	5.17E-24	10.84	4.50E-39	Glut9. 13. 15
Barhl2	TF	480 ± 92	6.7 ± 7.8	15.4 ± 2.38	6.02	9.86E-37	4.94	7.52E-53	nd
Slc17a6		47533 ± 1973	753 ± 360	748 ± 191	5.98	6.32E-51	5.98	2.37E-110	Glut1-15
Tlx3	TF	736 ± 64	13.7 ± 19.3	10.6 ± 9.6	5.78	1.63E-15	6.07	7.54E-69	expression low / Glut2-8, 10, 11, 14
Mgp		216.3 ± 16.7	4.5 ± 7.8	15 ± 16.2	5.6	4.43E-12	3.82	1.87E-08	ng
Adcyap1	NP	2469 ± 241	52 ± 32	73.3 ± 17.2	5.59	2.04E-42	5.07	8.12E-134	Glut2-8, 10,11,15
Nrn1	NF	20203 ± 1915	457 ± 164	458 ± 49	5.47	8.16E-86	5.45	1.18E-207	Glut1-15
Npff	NP	317 ± 26	7.7 ± 10.9	7.9 ± 7.1	5.43	6.03E-15	5.34	1.38E-33	Glut8, 9, 13, 15, Gaba8
Pou4f1	TF	1462 ± 131	33.9 ± 21.8	43 ± 10.3	5.4	7.59E-50	5.09	1.94E-113	Glut5, 13- 15
Ctla2a		104 ± 25	2.2 ± 3.8	8.1 ± 7.1	5.37	7.48E-07	3.69	3.89E-05	Glut5, 13- 15
Lhx9	TF	214.8 ± 23.5	5.1 ± 6.5	1.29 ± 1.56	5.37	6.79E-17	7.23	2.13E-34	nd
Pla2g5		507 ± 73	12.7 ± 22	16.2 ± 6.7	5.33	1.84E-05	4.98	9.34E-52	nd
Cacna2d1		6755 ± 656	170.2 ± 82	203 ± 46	5.32	8.04E-77	5.05	4.24E-175	Glut1-15
Vsx2	TF	1501 ± 91	41.9 ± 27.1	38.1 ± 6	5.17	6.07E-53	5.29	1.09E-132	nd
Nms	NP	217 ± 47	6.4 ± 11	1.48 ± 1.76	5.13	2.90E-07	6.96	2.35E-32	expression low / ng
Tmem163		4153 ± 563	122 ± 69	118 ± 13.3	5.1	1.45E-45	5.12	3.92E-176	nd
Phox2a	TF	136.9 ± 12.6	3.9 ± 6.7	3.4 ± 5.9	4.99	6.60E-07	5.17	4.54E-10	nd
Bdnf	NF	1097 ± 70	44 ± 36	18.5 ± 11.9	4.63	4.02E-15	5.88	9.28E-90	Glut1-3, 6, 7, 10, 11, 13-15
Nmur2	NPR	588 ± 84	24.9 ± 22.6	46 ± 38	4.59	6.13E-21	3.67	5.66E-16	Glut7, 8, 15
Cbln1	pNP	6341 ± 1471	310 ± 95	322 ± 53	4.36	1.17E-69	4.29	4.46E-110	Glut1-4, 8
Nmu	NP	571.9 ± 23.9	29 ± 34	7.6 ± 5.1	4.31	5.78E-06	6.17	3.25E-73	Glut1, 2-9, 11, 14
Chrm5		177 ± 28	8.8 ± 8.4	23.1 ± 13.1	4.27	3.14E-09	2.91	7.15E-09	expression low / Glut5, 7, 10, 11, Gaba1
Nmb	NP	204.9 ± 15.7	10.5 ± 8.2	4.5 ± 4.1	4.23	1.87E-12	5.51	1.08E-23	expression low / Glut7, 9, 11, 12, Gaba8
Tac2	NP	5416 ± 363	299 ± 86	536 ± 36	4.18	2.90E-85	3.33	7.35E-131	Glut1-11, 13, 14, Gaba4
Nts	NP	2582 ± 331	143 ± 86	130.6 ± 5.9	4.18	9.72E-31	4.29	9.74E-125	Glut1-9, 11, 13, 14, Gaba5
Lmx1b	TF	1745 ± 90	99.1 ± 24.9	62.9 ± 9.1	4.14	9.36E-78	4.77	2.02E-129	nd
Grpr	NPR	292 ± 34	17.2 ± 22	26.7 ± 11.6	4.04	6.37E-07	3.42	3.11E-21	Glut12-13
C1ql3		2262 ± 56	140 ± 22	171 ± 57	4	3.75E-94	3.71	5.63E-61	Glut1, 4-7, 9, 11, 13, Gaba3
Cpne4		2929 ± 473	186.8 ± 7.8	300 ± 40	3.97	1.07E-89	3.28	8.89E-71	Glut2
Lhx4	TF	249.1 ± 21.4	16.8 ± 17.9	25.3 ± 9.5	3.9	3.62E-06	3.29	2.32E-18	nd
Slc17a8		186 ± 28	12.5 ± 9.3	35.4 ± 15.6	3.86	2.31E-09	2.39	1.94E-07	ng
Cck	NP	7261 ± 778	538 ± 152	373 ± 74	3.76	4.13E-68	4.27	2.02E-140	Glut1-3, 5, 13-14
Mafa	TF	603 ± 78	46 ± 26	54.3 ± 40.2	3.71	3.26E-20	3.46	2.32E-17	nd
Prrxl1	TF	1436 ± 65	124 ± 59	180.8 ± 23.9	3.54	5.86E-34	2.98	9.05E-61	expression low / Glut3, 4, 6-11, 13
F2rl2		2146 ± 380	187 ± 156	264.1 ± 37.2	3.52	1.68E-12	3.01	1.04E-56	Glut1, 3-14, Gaba2
Ebf2	TF	3619 ± 436	355 ± 56	894.5 ± 59.7	3.35	8.47E-71	2.01	1.66E-40	Glut1-13, Gaba14, 15
Aldh1a2		179 ± 75	17.6 ± 11.8	17 ± 14.2	3.35	1.42E-07	3.35	1.94E-07	Glut14
Serpina3g		148 ± 36	15.8 ± 16.7	40.2 ± 8.1	3.23	8.13E-05	1.87	1.01E-04	nd
Nptx2		1325 ± 141	146.7 ± 24.5	181.2 ± 5.5	3.17	5.94E-48	2.86	1.79E-54	expression low / Glut2, 6, 10, 12- 14
Grp	NP	1085 ± 211	127 ± 39	105 ± 35	3.1	1.25E-28	3.36	2.48E-36	Glut5-12
Car12		423.4 ± 10.1	52.2 ± 9.1	71 ± 70	3.02	1.25E-23	2.56	1.64E-04	Glut6-9
Pou4f2		207 ± 39	25.7 ± 13.6	8.5 ± 4.4	3.01	1.79E-09	4.61	8.21E-21	ng
Sox14	TF	169 ± 27	21 ± 21.8	1.11 ± 1.92	3.01	2.32E-03	7.24	1.52E-24	nd
Tac1	NP	3017 ± 220	399 ± 92	848 ± 210	2.92	2.22E-48	1.82	1.09E-21	Glut4, 9-11, 14, Gaba9
Trh	NP	274 ± 44	37 ± 30	36.1 ± 16.6	2.88	1.43E-04	2.91	2.38E-14	Glut3, 4, Gaba6

Table S2 Most enriched genes in vGluT2 vs. vGAT. Group: TF = transcription factor, NP = neuropeptide, pNP = putative neuropeptide (classification of neuropeptides and putative neuropeptides is based on Burbach (2011)), NF = neurotrophic factor, NPR = neuropeptide receptor. sc data = single-cell data from Haring et al. (2018): nd = not detected, ng = not grouped.

		vGluT2 [norm. count]	vGAT [norm. count]	Gad67 [norm. count]	vGluT2 vs. vGAT		vGluT2 vs. Gad67		
Gene	Group	Mean ± Stddev	Mean ± Stddev	Mean ± Stddev	log2 Ratio	FDR	log2 Ratio	FDR	
Tfap2b	TF	30.6 ± 17.1	1455 ± 48	717 ± 60	-5.58	2.29E-83	-4.57	1.97E-48	Gaba10, 13
Pax8	TF	63.7 ± 9	2726 ± 338	3115 ± 419	-5.43	1.32E-140	-5.63	1.05E-171	Gaba2-8, 11, 12
Sall3	TF	201 ± 63	5381 ± 754	6224 ± 804	-4.75	3.76E-92	-4.97	5.74E-122	Gaba2-15
Slc32a1		734 ± 397	19147 ± 2462	20990 ± 2384	-4.71	9.09E-39	-4.85	1.12E-45	Gaba1-15
Ighg3		0.7 ± 1.21	20 ± 3	124 ± 11.5	-4.69	1.24E-01	-7.39	8.67E-18	nd
Gad2		802 ± 111	18347 ± 3188	26077 ± 1196	-4.52	4.58E-91	-5.04	5.70E-156	Gaba1-15
Gad1		823 ± 140	18310 ± 4760	40767 ± 3643	-4.48	1.69E-70	-5.64	1.19E-127	Gaba1-15
Pax2	TF	321 ± 168	6971 ± 499	7070 ± 544	-4.45	3.30E-50	-4.48	8.72E-56	Gaba2-15
Pnoc	NP	116 ± 30	2499 ± 184	3953 ± 558	-4.44	7.27E-92	-5.11	2.70E-129	Gaba1-15
Lhx5	TF	59.4 ± 14.7	1129 ± 164	1129 ± 121	-4.24	6.52E-59	-4.24	3.88E-73	Gaba3, 4, 7, 8, 11-14
Gata3	TF	6.8 ± 6.2	128 ± 45	18 ± 3.5	-4.18	2.17E-06	-1.39	6.05E-01	ng
Slc6a5		1838 ± 823	32313 ± 2418	33567 ± 5237	-4.14	3.30E-35	-4.2	4.87E-43	Gaba14
Gbx1	TF	67.3 ± 19.2	1172 ± 90	1359 ± 102	-4.14	3.10E-58	-4.36	6.33E-80	expression low / Gaba2-4, 6-13
Tal1	TF	11.8 ± 6.4	203.6 ± 11	21 ± 22.2	-4.06	9.14E-15	-0.83	7.89E-01	ng
Dmkn		5.6 ± 7.9	90.9 ± 12.5	123 ± 28	-4.04	1.31E-03	-4.49	1.13E-06	expression low / Gaba2, 3, 6, 10, 12
Pax5	TF	13.4 ± 9.9	203 ± 66	204 ± 121	-3.91	1.47E-10	-3.92	4.29E-10	nd
Aqp6		6.6 ± 7.4	78 ± 51	174 ± 32	-3.57	2.34E-02	-4.77	8.88E-12	Gaba14, 15
Dmrt3	TF	16.2 ± 17.6	189 ± 22.2	112 ± 29	-3.57	7.82E-06	-2.81	2.75E-03	nd
Pkd1l2		13.5 ± 5.3	159 ± 27	0 ± 0	-3.53	2.11E-09	6.84	1.80E-02	expression low / ng
Slc6a1		1513 ± 451	15617 ± 4052	26070 ± 3382	-3.37	1.05E-37	-4.12	2.78E-69	nd
Cdh3		64 ± 29	651 ± 253	1336 ± 184	-3.36	4.75E-17	-4.41	3.68E-58	expression low / Glut2, Gaba3, Gaba8, Gaba10-12, 14
Grb7		7.3 ± 6.9	73 ± 36	84 ± 38	-3.33	2.46E-02	-3.54	1.31E-03	nd
Gata2	TF	11.6 ± 9.5	109 ± 36	12.5 ± 10.3	-3.23	3.46E-04	-0.11	9.77E-01	nd
Pkd2l1		41 ± 30	368 ± 110	30.4 ± 20.6	-3.17	3.57E-11	0.42	8.20E-01	nd
Alox12b		15.2 ± 12.5	133 ± 72	202 ± 105	-3.13	2.46E-04	-3.73	8.08E-09	Gaba14, 15
Htr3a		90.8 ± 21.7	752 ± 124	428 ± 75	-3.05	1.11E-28	-2.25	1.52E-15	Gaba10, 13-15
Asic4		63 ± 42	495 ± 31	610 ± 58	-2.99	2.27E-13	-3.3	1.25E-18	Gaba14
Slc30a3		585 ± 201	4564 ± 487	4491 ± 486	-2.97	6.37E-40	-2.96	1.78E-44	Glut1, 3-4, Gaba1,2, 4-7, 10-13
Ntsr1	NPR	63.3 ± 15.9	480 ± 34	726 ± 64	-2.92	1.85E-22	-3.53	1.27E-45	Gaba5, 14, 15
Actr5		58.4 ± 21.8	439 ± 81	65.4 ± 19.3	-2.9	1.60E-16	-0.16	9.00E-01	expression low / Gaba8-10, 13
Gpc4		42.3 ± 17	311 ± 48	602 ± 150	-2.89	3.30E-14	-3.85	1.86E-32	expression low / Gaba2-4, 9, 11-14
Shisa3		38.7 ± 22.9	273 ± 26	246 ± 64	-2.84	1.66E-10	-2.69	1.33E-09	nd
Klhl14		207.1 ± 5.1	1475 ± 154	1121 ± 4.4	-2.83	8.97E-48	-2.45	1.58E-42	Gaba15
Neurod2	TF	259.8 ± 8.5	1842 ± 146	1826 ± 92	-2.83	2.00E-55	-2.82	7.20E-70	Gaba3, 11, 14, 15
Krt25		59.2 ± 3.9	414 ± 98	650 ± 196	-2.81	1.40E-18	-3.47	2.26E-34	Gaba12
Lhx1	TF	154 ± 54	1063 ± 169	1133 ± 106	-2.79	8.26E-25	-2.89	6.51E-33	Gaba5-10, 12, 13
Neurod6	TF	141 ± 76	918 ± 132	659 ± 107	-2.72	1.94E-15	-2.24	3.56E-11	Gaba2-3, 10, 12-15
C130060K24Rik		14.5 ± 8.2	94 ± 67	93 ± 34	-2.68	1.37E-02	-2.67	7.61E-04	nd
Neurod1	TF	63 ± 26	391 ± 72	379 ± 58	-2.64	7.82E-13	-2.6	7.23E-15	Gaba11, 14, 15
Lgals9		219 ± 39	1343 ± 160	1813 ± 243	-2.62	5.16E-34	-3.06	3.38E-59	Gaba1-15
Ghsr		43.4 ± 22.2	263 ± 57	255 ± 48	-2.62	5.63E-09	-2.58	2.60E-10	ng
Rasgrp1		237 ± 27	1256 ± 300	1337 ± 195	-2.41	3.56E-25	-2.5	9.85E-40	Gaba1-15
Erich2		7 ± 6.8	37.3 ± 14.9	154 ± 30	-2.39	3.52E-01	-4.43	4.02E-11	ng
Islr2		32.1 ± 3.1	163 ± 68	248 ± 33	-2.35	4.53E-05	-2.96	4.51E-18	Gaba5-7, 12
Fblim1		42.2 ± 19.1	214 ± 28	179 ± 35	-2.35	2.52E-07	-2.1	4.24E-06	ng
Irx1	TF	23.7 ± 21.4	111.2 ± 20.4	216 ± 52	-2.25	1.30E-01	-3.21	2.28E-04	ng
Nrl	TF	28 ± 34	130 ± 31	127 ± 45	-2.21	4.22E-02	-2.18	2.00E-02	Gaba8, 9
Kcnf1		102.2 ± 24.4	466 ± 55	685 ± 156	-2.19	1.02E-13	-2.76	2.02E-25	Gaba10, 12, 14
Gbx2	TF	88.7 ± 14.1	399 ± 119	348 ± 84	-2.18	1.95E-10	-1.99	2.18E-11	Gaba10
Irx2	TF	23.1 ± 4.2	102 ± 50	244.3 ± 22	-2.14	1.06E-02	-3.4	3.21E-21	ng

Table S3 Most enriched genes in vGAT vs. vGluT2. Group: TF = transcription factor, NP = neuropeptide, pNP = putative neuropeptide (classification of neuropeptides and putative neuropeptides is based on Burbach (2011)), NF = neurotrophic factor, NPR = neuropeptide receptor. sc data = single-cell data from Haring et al. (2018): nd = not detected, ng = not grouped.

		vGluT2 [norm. count]	vGAT [norm. count]	Gad67 [norm. count]	vGluT2 vs. vGAT		vGluT2 vs. Gad67		neuronal cell type
Gene	Group	Mean ± Stddev	Mean ± Stddev	Mean ± Stddev	log2 Ratio	FDR	log2 Ratio	FDR	sc Data
Barhl1	TF	382 ± 38	4.3 ± 2.9	0 ± 0	6.4	3.25E-49	11.65	5.24E-74	nd
Crh	NP	219 ± 44	3 ± 2.6	0 ± 0	6.05	5.17E-24	10.84	4.50E-39	Glut9, 13, 15
Lhx2	TF	199 ± 35	0.96 ± 1.67	0 ± 0	7.62	9.29E-26	10.71	2.02E-35	ng
Sox14	TF	169 ± 27	21 ± 21.8	1.11 ± 1.92	3.01	2.32E-03	7.24	1.52E-24	nd
Lhx9	TF	214.8 ± 23.5	5.1 ± 6.5	1.29 ± 1.56	5.37	6.79E-17	7.23	2.13E-34	nd
Nms	NP	217 ± 47	6.4 ± 11	1.48 ± 1.76	5.13	2.90E-07	6.96	2.35E-32	expression low / ng
Pkd1l2		13.5 ± 5.3	159 ± 27	0 ± 0	-3.53	2.11E-09	6.84	1.80E-02	expression low / ng
Nmu	NP	571.9 ± 23.9	29 ± 34	7.6 ± 5.1	4.31	5.78E-06	6.17	3.25E-73	Glut1, 3-9,11, 14
Tlx3	TF	736 ± 64	13.7 ± 19.3	10.6 ± 9.6	5.78	1.63E-15	6.07	7.54E-69	expression low / Glut2-8, 10, 11, 14
Slc17a6		47533 ± 1973	753 ± 360	748 ± 191	5.98	6.32E-51	5.98	2.37E-110	Glut1-15
Bdnf	NF	1097 ± 70	44 ± 36	18.5 ± 11.9	4.63	4.02E-15	5.88	9.28E-90	Glut1-3, 6, 7, 10, 11, 13-15
Nmb	NP	204.9 ± 15.7	10.5 ± 8.2	4.5 ± 4.1	4.23	1.87E-12	5.51	1.08E-23	expression low / Glut7, 9, 11, 12, Gaba8
Nrn1	NF	20203 ± 1915	457 ± 164	458.1 ± 48.8	5.47	8.16E-86	5.45	1.18E-207	Glut1-15
Npff	NP	317 ± 26	7.7 ± 10.9	7.9 ± 7.1	5.43	6.03E-15	5.34	1.38E-33	Glut8, 9, 13, 15, Gaba8
Vsx2	TF	1501 ± 91	42 ± 27	38.1 ± 6	5.17	6.07E-53	5.29	1.09E-132	nd
Phox2a	TF	136.9 ± 12.6	3.9 ± 6.7	3.4 ± 5.9	4.99	6.60E-07	5.17	4.54E-10	nd
Tmem163		4153 ± 563	122 ± 69	118 ± 13.3	5.1	1.45E-45	5.12	3.92E-176	ng
Pou4f1	TF	1462 ± 131	33.9 ± 21.8	43 ± 10.3	5.4	7.59E-50	5.09	1.94E-113	Glut5, 13-15
Adcyap1	NP	2469 ± 241	52 ± 32	73.3 ± 17.2	5.59	2.04E-42	5.07	8.12E-134	Glut1, 3-11, 13-15
Cacna2d1		6755 ± 656	170 ± 82	202.6 ± 46.4	5.32	8.04E-77	5.05	4.24E-175	Glut1-15, Gaba1
Pla2g5		507 ± 73	12.7 ± 22	16.2 ± 6.7	5.33	1.84E-05	4.98	9.34E-52	Glut1, 2, 4, 5
Barhl2	TF	480 ± 92	6.7 ± 7.8	15.4 ± 2.38	6.02	9.86E-37	4.94	7.52E-53	nd
Shox2	TF	765 ± 32	11.5 ± 10.4	25.6 ± 20.9	6.12	7.88E-35	4.91	9.81E-21	nd
Lmx1b	TF	1745 ± 90	99.1 ± 24.9	62.9 ± 9.1	4.14	9.36E-78	4.77	2.02E-129	nd
Pou4f2	TF	207 ± 39	25.7 ± 13.6	8.5 ± 4.4	3.01	1.79E-09	4.61	8.21E-21	ng
Nts	NP	2582 ± 331	143 ± 86	130.6 ± 5.9	4.18	9.72E-31	4.29	9.74E-125	Glut1-9, 11, 13, 14, Gaba5
Cbln1	pNP	6341 ± 1471	310 ± 95	322 ± 53	4.36	1.17E-69	4.29	4.46E-110	Glut1-4, 8
Cck	NP	7261 ± 778	538 ± 152	373 ± 74	3.76	4.13E-68	4.27	2.02E-140	Glut1-3, 5, 13, 14
Ucn3	NP	106 ± 34	0 ± 0	5.9 ± 10.2	9.87	8.52E-14	4.18	4.60E-04	Glut3
Vip	NP	171 ± 38	34.5 ± 7	10.4 ± 11.8	2.3	5.98E-06	4.05	1.50E-07	ng
Dmrt2	TF	151.9 ± 2	29.9 ± 23.8	9.5 ± 6.9	2.32	8.58E-04	3.97	2.93E-11	ng
Olig3	TF	176 ± 28	1.6 ± 1.9	12.2 ± 7.1	6.58	5.80E-20	3.82	4.92E-13	nd
Mgp		216.3 ± 16.7	4.5 ± 7.8	15 ± 16.2	5.6	4.43E-12	3.82	1.87E-08	ng
C1ql3		2262 ± 56	140 ± 22	171 ± 57	4	3.75E-94	3.71	5.63E-61	Glut1, 4-7, 9, 11, 13, Gaba3
Ctla2a		104 ± 25	2.2 ± 3.8	8.1 ± 7.1	5.37	7.48E-07	3.69	3.89E-05	ng
Nmur2	NPR	588 ± 84	24.9 ± 22.6	46 ± 38	4.59	6.13E-21	3.67	5.66E-16	Glut7, 8, 15
Crhbp		435 ± 66	108.2 ± 6.7	33.7 ± 5.9	2.01	2.39E-12	3.67	9.56E-36	Glut7, 8
Clic6		217.2 ± 9.4	59 ± 43	18.1 ± 8	1.88	1.45E-03	3.58	3.01E-17	ng
Mafa	TF	603 ± 78	46 ± 26	54 ± 40	3.71	3.26E-20	3.46	2.32E-17	nd
Grpr	NPR	292 ± 34	17.2 ± 22	26.7 ± 11.6	4.04	6.37E-07	3.42	3.11E-21	Glut12-13
Grp	NP	1085 ± 211	127 ± 39	105 ± 35	3.1	1.25E-28	3.36	2.48E-36	Glut5-12
Aldh1a2		179 ± 75	17.6 ± 11.8	17 ± 14.2	3.35	1.42E-07	3.35	1.94E-07	Glut14
Tac2	NP	5416 ± 363	299 ± 86	536 ± 36	4.18	2.90E-85	3.33	7.35E-131	Glut1-11, 13, 14, Gaba4
Lhx4	TF	249.1 ± 21.4	16.8 ± 17.9	25.3 ± 9.5	3.9	3.62E-06	3.29	2.32E-18	nd
Cpne4		2929 ± 473	186.8 ± 7.8	300 ± 40	3.97	1.07E-89	3.28	8.89E-71	Glut2
Rprm		511 ± 76	124 ± 82	57.6 ± 17.8	2.05	3.88E-06	3.14	2.48E-27	Glut3-8, 14, 15
Ctxn3		189 ± 54	124 ± 41	21.5 ± 3.2	0.6	5.02E-01	3.12	3.64E-12	ng
F2rl2		2146 ± 380	187 ± 156	264 ± 37	3.52	1.68E-12	3.01	1.04E-56	Glut1, 3-14, Gaba2
Prrxl1	TF	1436 ± 65	124 ± 59	180.8 ± 23.9	3.54	5.86E-34	2.98	9.05E-61	expression low / Glut3, 4, 6-11, 13
Gm10684		316 ± 55	78 ± 54	40.5 ± 4.4	2.01	3.10E-04	2.95	3.43E-21	ng

Table S4 Most enriched genes in vGluT2 vs. Gad67. Group: TF = transcription factor, NP = neuropeptide, pNP = putative neuropeptide (classification of neuropeptides and putative neuropeptides is based on Burbach (2011)), NF = neurotrophic factor, NPR = neuropeptide receptor. sc data = single-cell data from Haring et al. (2018): nd = not detected, ng = not grouped.

		vGluT2 [norm. count]	vGAT [norm. count]	Gad67 [norm. count]	vGluT2 vs. vGAT		vGluT2 vs. Gad67		neuronal cell type
Gene	Group	Mean ± Stddev	Mean ± Stddev	Mean ± Stddev	log2 Ratio	FDR	log2 Ratio	FDR	sc Data
Ighg3		0.7 ± 1.21	20.0 ± 3.0	124 ± 11.5	-4.69	1.24E-01	-7.39	8.67E-18	nd
Gad1		823 ± 140	18310 ± 4760	40767 ± 3643	-4.48	1.69E-70	-5.64	1.19E-127	Gaba1-15
Pax8	TF	63.7 ± 9	2726 ± 338	3115 ± 419	-5.43	1.32E-140	-5.63	1.05E-171	Gaba2-8, 11, 12
Chrna2		16.4 ± 14.6	9.28 ± 0.51	606 ± 100	0.8	9.30E-01	-5.26	1.09E-16	nd
Pnoc	NP	116 ± 30	2499 ± 184	3953 ± 558	-4.44	7.27E-92	-5.11	2.70E-129	Gaba1-15
Gad2		802 ± 111	18347 ± 3188	26077 ± 1196	-4.52	4.58E-91	-5.04	5.70E-156	Gaba1-15
Sall3	TF	201 ± 63	5381 ± 754	6224 ± 804	-4.75	3.76E-92	-4.97	5.74E-122	Gaba2-15
Slc32a1		734 ± 397	19147 ± 2462	20990 ± 2384	-4.71	9.09E-39	-4.85	1.12E-45	Gaba1-15
Aqp6		6.6 ± 7.4	78 ± 51	174 ± 32	-3.57	2.34E-02	-4.77	8.88E-12	Gaba14, 15
Tfap2b	TF	30.6 ± 17.1	1455 ± 48	717 ± 60	-5.58	2.29E-83	-4.57	1.97E-48	Gaba10, 13
Dmkn		5.6 ± 7.9	90.9 ± 12.5	123 ± 28	-4.04	1.31E-03	-4.49	1.13E-06	expression low / Gaba2, 3, 6, 10, 12
Pax2	TF	321 ± 168	6971 ± 499	7070 ± 544	-4.45	3.30E-50	-4.48	8.72E-56	Gaba2-15
Erich2		7 ± 6.8	37.3 ± 14.9	154 ± 30	-2.39	3.52E-01	-4.43	4.02E-11	ng
Cdh3		64 ± 29	651 ± 253	1336 ± 184	-3.36	4.75E-17	-4.41	3.68E-58	expression low / Glut2, Gaba3, Gaba8, Gaba10-12, 14
Gbx1	TF	67.3 ± 19.2	1172 ± 90	1359 ± 102	-4.14	3.10E-58	-4.36	6.33E-80	expression low / Gaba2-4, 6-13
Lhx5		59.4 ± 14.7	1129 ± 164	1129 ± 121	-4.24	6.52E-59	-4.24	3.88E-73	Gaba3, 4, 7, 8, 11-14
Slc6a5		1838 ± 823	32313 ± 2418	33567 ± 5237	-4.14	3.30E-35	-4.2	4.87E-43	Gaba14
Slc6a1		1513 ± 451	15617 ± 4052	26070 ± 3382	-3.37	1.05E-37	-4.12	2.78E-69	ng
Pax5	TF	13.4 ± 9.9	203 ± 66	204 ± 121	-3.91	1.47E-10	-3.92	4.29E-10	nd
Gpc4		42.3 ± 17	311 ± 48	602 ± 150	-2.89	3.30E-14	-3.85	1.86E-32	expression low / Gaba2-4, 9, 11-14
Alox12b		15.2 ± 12.5	133 ± 72	202 ± 105	-3.13	2.46E-04	-3.73	8.08E-09	Gaba14, 15
Grb7		7.3 ± 6.9	73 ± 36	84 ± 38	-3.33	2.46E-02	-3.54	1.31E-03	ng
Ntsr1	NPR	63.3 ± 15.9	480 ± 34	726 ± 64	-2.92	1.85E-22	-3.53	1.27E-45	Gaba5, 14, 15
Krt25		59.2 ± 3.9	414 ± 98	650 ± 196	-2.81	1.40E-18	-3.47	2.26E-34	Gaba12
Irx2	TF	23.1 ± 4.2	102 ± 50	244.3 ± 22	-2.14	1.06E-02	-3.4	3.21E-21	ng
Slc12a7		21.2 ± 13.7	90 ± 50	215.3 ± 7.2	-2.08	7.18E-02	-3.35	4.56E-13	Gaba14
Asic4		63 ± 42	495 ± 31	610 ± 58	-2.99	2.27E-13	-3.3	1.25E-18	Gaba14
Irx1	TF	23.7 ± 21.4	111.2 ± 20.4	216 ± 52	-2.25	1.30E-01	-3.21	2.28E-04	ng
Otp	TF	112 ± 92	429 ± 96	967 ± 82	-1.95	3.03E-04	-3.13	6.67E-13	nd
Lgals9		219 ± 39	1343 ± 160	1813 ± 243	-2.62	5.16E-34	-3.06	3.38E-59	Gaba1-15
Adamtsl2		19.8 ± 2.7	58 ± 26	154 ± 58	-1.56	3.22E-01	-2.97	8.24E-09	Gaba4
Islr2		32.1 ± 3.1	163 ± 68	248 ± 33	-2.35	4.53E-05	-2.96	4.51E-18	Gaba5-7, 12
Slc30a3		585 ± 201	4564 ± 487	4491 ± 486	-2.97	6.37E-40	-2.96	1.78E-44	Glut1, 3-4, Gaba1,2, 4-7, 10-13
Crabp1		689 ± 32	2431 ± 336	5210 ± 543	-1.82	4.04E-25	-2.93	2.70E-101	Glut9, Gaba2, 3, 7, 8, 14, 15
Lhx1	TF	154 ± 54	1063 ± 169	1133 ± 106	-2.79	8.26E-25	-2.89	6.51E-33	Gaba5-10, 12, 13
Ppef1		19.2 ± 10.3	64.9 ± 8.7	140 ± 32	-1.75	2.12E-01	-2.86	1.11E-06	Gaba11, 14
Nfatc1	TF	32.1 ± 14.3	135 ± 67	222.9 ± 11	-2.09	7.21E-03	-2.83	8.07E-12	Gaba14, 15
Neurod2	TF	259.8 ± 8.5	1842 ± 146	1826 ± 92	-2.83	2.00E-55	-2.82	7.20E-70	Gaba3, 11, 14, 15
Dmrt3	TF	16.2 ± 17.6	189 ± 22.2	112 ± 29	-3.57	7.82E-06	-2.81	2.75E-03	nd
Kcnf1		102.2 ± 24.4	466 ± 55	685 ± 156	-2.19	1.02E-13	-2.76	2.02E-25	Gaba10, 12, 14
Shisa3		38.7 ± 22.9	273 ± 26	246 ± 64	-2.84	1.66E-10	-2.69	1.33E-09	ng
C130060K24Rik		14.5 ± 8.2	94 ± 67	93 ± 34	-2.68	1.37E-02	-2.67	7.61E-04	ng
Neurod1	TF	63 ± 26	391 ± 72	379 ± 58	-2.64	7.82E-13	-2.6	7.23E-15	Gaba11, 14, 15
En1	TF	47.1 ± 11.6	164 ± 30	281 ± 119	-1.8	5.41E-04	-2.59	2.92E-11	nd
Ghsr		43.4 ± 22.2	263 ± 57	255 ± 48	-2.62	5.63E-09	-2.58	2.60E-10	ng
Rasgrp1		237 ± 27	1256 ± 300	1337 ± 195	-2.41	3.56E-25	-2.5	9.85E-40	Gaba1-15
Irgm1		127.8 ± 12.4	559 ± 115	715 ± 124	-2.13	2.50E-14	-2.49	5.19E-27	expression low / ng
Klhl14		207.1 ± 5.1	1475 ± 154	1121 ± 4.4	-2.83	8.97E-48	-2.45	1.58E-42	Gaba15
Pdyn	NP	384 ± 77	966 ± 280	2075 ± 385	-1.33	1.94E-06	-2.44	1.46E-33	Gaba3-5
Npy	NP	1262 ± 325	3605 ± 812	6622 ± 880	-1.52	1.24E-10	-2.41	3.66E-38	Gaba1-15

Table S5 Most enriched genes in Gad67 vs. vGluT2. Group: TF = transcription factor, NP = neuropeptide, pNP = putative neuropeptide (classification of neuropeptides and putative neuropeptides is based on Burbach (2011)), NF = neurotrophic factor, NPR = neuropeptide receptor. sc data = single-cell data from Haring et al. (2018): nd = not detected, ng = not grouped.

Neurotransmitter receptor expression

Glutamate receptors

	vGluT2 [norm. count]	vGAT [norm. count]	Gad67 [norm. count]	vGluT2 vs. vGAT		vGluT2 vs. Gad67	
**Gene**	**Mean ± Stddev**	**Mean ± Stddev**	**Mean ± Stddev**	**log2 Ratio**	**FDR**	**log2 Ratio**	**FDR**
Gria1	3853 ± 429	6189 ± 1252	9054 ± 821	-0.69	**1.92E-03**	**-1.24**	**4.49E-19**
Gria2	7524 ± 295	5010 ± 579	6896 ± 1352	0.59	**2.98E-03**	0.11	7.16E-01
Gria3	3327 ± 203	3423 ± 208	3229 ± 304	-0.04	9.66E-01	0.03	9.17E-01
Gria4	4879 ± 727	4476 ± 238	4437 ± 544	0.12	8.68E-01	0.13	6.66E-01
Grik1	200 ± 56	465 ± 183	453 ± 94	**-1.22**	**2.32E-03**	**-1.19**	**8.15E-05**
Grik2	1074 ± 299	2411 ± 362	2676 ± 353	**-1.17**	**3.08E-07**	**-1.33**	**7.94E-11**
Grik3	737 ± 97	2753 ± 114	3288 ± 275	**-1.90**	**9.00E-30**	**-2.17**	**2.74E-44**
Grik4	651 ± 67	721 ± 179	735 ± 44	-0.15	8.97E-01	-0.19	5.66E-01
Grik5	2341 ± 392	2481 ± 229	2289 ± 396	-0.09	9.28E-01	0.02	9.66E-01
Grin1	16293 ± 2625	14053 ± 2385	17130 ± 3003	0.21	8.29E-01	-0.08	8.54E-01
Grin2a	3811 ± 484	3237 ± 352	3463 ± 703	0.23	5.72E-01	0.13	7.07E-01
Grin2b	4048 ± 192	3630 ± 254	4605 ± 535	0.16	7.71E-01	-0.20	3.65E-01
Grin2c	41.4 ± 18.9	97.4 ± 21.4	116.4 ± 7.8	**-1.24**	2.43E-01	**-1.51**	**1.25E-02**
Grin2d	1775 ± 178	2062 ± 121	2485 ± 454	-0.22	6.25E-01	-0.50	**1.75E-02**
Grin3a	3791 ± 271	1965 ± 135	2127 ± 174	0.95	**1.79E-09**	0.82	**2.86E-09**
Grin3b	47.5 ± 11.5	49.0 ± 33.0	58.9 ± 26.6	-0.06	9.92E-01	-0.32	8.08E-01

Table S6 Ionotropic glutamate receptors (AMPA, kainate and NMDA receptors). Significant (FDR ≤ 0.05) and |log2 ratio| ≥ 1 enrichment values are highlighted in **bold**.

	vGluT2 [norm. count]	vGAT [norm. count]	Gad67 [norm. count]	vGluT2 vs. vGAT		vGluT2 vs. Gad67	
**Gene**	**Mean ± Stddev**	**Mean ± Stddev**	**Mean ± Stddev**	**log2 Ratio**	**FDR**	**log2 Ratio**	**FDR**
Grm1	2407 ± 156	1632 ± 116	1912 ± 419	0.56	**5.76E-03**	0.32	2.15E-01
Grm2	157.4 ± 23.5	184 ± 109	315.9 ± 8.2	-0.22	9.27E-01	**-1.02**	**2.70E-04**
Grm3	424 ± 79	946 ± 237	1243 ± 198	**-1.16**	**1.86E-05**	**-1.57**	**1.06E-14**
Grm4	2116 ± 315	1574 ± 110	1714 ± 263	0.43	1.03E-01	0.29	2.55E-01
Grm5	5322 ± 309	7181 ± 1239	9795 ± 1607	-0.43	1.07E-01	-0.89	**2.53E-08**
Grm6	0 ± 0	0 ± 0	0 ± 0	0.00	NA	0.00	NA
Grm7	1559 ± 184	1732 ± 161	1985 ± 194	-0.15	8.25E-01	-0.36	9.15E-02
Grm8	420 ± 56	447 ± 29	549 ± 176	-0.09	9.48E-01	-0.40	3.07E-01

Table S7 Metabotropic glutamate receptors. Significant (FDR ≤ 0.05) |log2 ratio| ≥ 1 enrichment values are highlighted in **bold**. NA = not applicable.

GABA receptors

	vGluT2 [norm. count]	vGAT [norm. count]	Gad67 [norm. count]	vGluT2 vs. vGAT		vGluT2 vs. Gad67	
**Gene**	**Mean ± Stddev**	**Mean ± Stddev**	**Mean ± Stddev**	**log2 Ratio**	**FDR**	**log2 Ratio**	**FDR**
Gabra1	4236 ± 493	4361 ± 357	5504 ± 592	-0.04	9.68E-01	-0.39	**2.87E-02**
Gabra2	977 ± 83	643 ± 93	638.4 ± 19.4	0.60	**2.39E-02**	0.60	**1.76E-03**
Gabra3	5357 ± 498	4949 ± 585	6146 ± 837	0.11	8.84E-01	-0.21	3.57E-01
Gabra4	653 ± 39	555 ± 63	530.4 ± 22.5	0.23	7.36E-01	0.29	3.08E-01
Gabra5	5260 ± 959	2574 ± 84	2355 ± 322	**1.03**	**2.11E-09**	**1.15**	**3.97E-12**
Gabra6	0 ± 0	0 ± 0	0 ± 0	0.00	NA	0.00	NA
Gabrb1	2706 ± 238	2797 ± 353	3234 ± 418	-0.05	9.64E-01	-0.27	2.01E-01
Gabrb2	1637 ± 81	1152 ± 94	1364 ± 183	0.51	**2.12E-02**	0.25	3.06E-01
Gabrb3	11663 ± 1308	9704 ± 1598	10398 ± 1382	0.26	6.50E-01	0.15	6.18E-01
Gabrg1	593.2 ± 16.6	511 ± 119	941 ± 211	0.21	8.11E-01	-0.68	**4.50E-03**
Gabrg2	6631 ± 484	5536 ± 958	7043 ± 1243	0.26	5.52E-01	-0.10	7.60E-01
Gabrg3	1553 ± 158	1431 ± 452	1736 ± 237	0.12	9.24E-01	-0.17	5.58E-01
Gabrd	36.2 ± 11.0	40 ± 52	28.8 ± 6.0	-0.13	9.89E-01	0.32	8.51E-01
Gabre	97.6 ± 7.8	44.1 ± 11.7	58.7 ± 24.2	**1.14**	2.46E-01	0.72	3.44E-01
Gabrp	0 ± 0	0 ± 0	4.1 ± 7.0	0.00	NA	**-5.13**	NA
Gabrq	538.8 ± 24.7	217 ± 71	330 ± 85	**1.31**	**2.06E-05**	0.69	**1.65E-02**
Gabrr1	12.1 ± 11.5	0.32 ± 0.56	0 ± 0	**4.85**	3.62E-01	**6.68**	6.79E-02
Gabrr2	8.1 ± 8.3	17.6 ± 15.3	8.3 ± 9.1	**-1.13**	9.06E-01	-0.06	NA
Gabrr3	0 ± 0	0 ± 0	0 ± 0	0.00	NA	0.00	NA

Table S8 Ionotropic GABA receptors (GABA$_A$ and –ρ receptor). Significant (FDR ≤ 0.05) and |log2 ratio| ≥ 1 enrichment values are highlighted in **bold**. NA = not applicable.

	vGluT2 [norm. count]	vGAT [norm. count]	Gad67 [norm. count]	vGluT2 vs. vGAT		vGluT2 vs. Gad67	
**Gene**	**Mean ± Stddev**	**Mean ± Stddev**	**Mean ± Stddev**	**log2 Ratio**	**FDR**	**log2 Ratio**	**FDR**
Gabbr1	26433 ± 4708	26183 ± 3572	28147 ± 4127	0.01	9.96E-01	-0.10	8.39E-01
Gabbr2	10007 ± 784	9451 ± 171	10552 ± 1623	0.08	9.36E-01	-0.09	8.01E-01

Table S9 Metabotropic GABA receptors (GABA$_B$). Significant (FDR ≤ 0.05) and |log2 ratio| ≥ 1 enrichment values are highlighted in **bold**.

Glycine receptors

	vGluT2 [norm. count]	vGAT [norm. count]	Gad67 [norm. count]	vGluT2 vs. vGAT		vGluT2 vs. Gad67	
**Gene**	**Mean ± Stddev**	**Mean ± Stddev**	**Mean ± Stddev**	**log2 Ratio**	**FDR**	**log2 Ratio**	**FDR**
Glra1	13117 ± 927	9331 ± 786	9775 ± 1424	0.49	8.37E-02	0.41	5.40E-02
Glra2	1320 ± 213	1348 ± 353	1382 ± 182	-0.03	9.83E-01	-0.08	8.39E-01
Glra3	710 ± 77	545 ± 86	667 ± 66	0.38	3.95E-01	0.08	8.45E-01
Glra4	30.9 ± 13.8	20.8 ± 11.3	30.5 ± 13.1	0.57	9.17E-01	0.01	9.98E-01
Glrb	19773 ± 2483	17713 ± 2342	17187 ± 2695	0.16	9.01E-01	0.19	6.08E-01

Table S10 Glycine receptors. Significant (FDR ≤ 0.05) and |log2 ratio| ≥ 1 enrichment values are highlighted in **bold**.

Acetylcholine receptors

	vGluT2 [norm. count]	vGAT [norm. count]	Gad67 [norm. count]	vGluT2 vs. vGAT		vGluT2 vs. Gad67	
**Gene**	**Mean ± Stddev**	**Mean ± Stddev**	**Mean ± Stddev**	**log2 Ratio**	**FDR**	**log2 Ratio**	**FDR**
Chrna1	4.5 ± 7.8	3.8 ± 6.7	0 ± 0	0.22	NA	**5.27**	NA
Chrna2	16.4 ± 14.6	9.3 ± 0.5	606 ± 100	0.80	9.30E-01	**-5.26**	**1.09E-16**
Chrna3	117 ± 39	211 ± 53	420.9 ± 15.4	-0.86	1.71E-01	**-1.87**	**4.08E-10**
Chrna4	4316 ± 654	3537 ± 467	3457 ± 643	0.29	4.33E-01	0.31	1.95E-01
Chrna5	26.2 ± 16.7	8.9 ± 8.5	97.6 ± 22.5	**1.56**	7.82E-01	**-1.92**	**1.79E-02**
Chrna6	89.1 ± 43.0	3.3 ± 2.9	0 ± 0	**4.66**	**1.59E-05**	**9.54**	**1.05E-12**
Chrna7	513 ± 114	493 ± 72	674 ± 239	0.05	9.75E-01	-0.41	3.58E-01
Chrna9	0 ± 0	0 ± 0	3.0 ± 5.2	0.00	NA	**-4.73**	NA
Chrna10	0 ± 0	3.9 ± 6.7	6.6 ± 6.5	**-5.13**	NA	**-5.82**	NA
Chrnb1	4.3 ± 7.4	0 ± 0	0 ± 0	**5.28**	NA	**5.21**	NA
Chrnb2	1921 ± 238	1820 ± 286	2018 ± 214	0.08	9.43E-01	-0.08	8.08E-01
Chrnb3	38.1 ± 14.1	14.0 ± 11.3	8.5 ± 7.9	**1.42**	6.81E-01	**2.15**	2.25E-01
Chrnb4	11.0 ± 3.7	58 ± 28	95.5 ± 21.1	**-2.39**	8.09E-02	**-3.14**	**8.46E-06**
Chrnd	0 ± 0	0 ± 0	0 ± 0	0.00	NA	0.00	NA
Chrne	0 ± 0	0 ± 0	0 ± 0	0.00	NA	0.00	NA
Chrng	3.8 ± 6.6	0 ± 0	0 ± 0	**5.10**	NA	**5.03**	NA

Table S11 Ionotropic acetylcholine receptors (nicotinic). Significant (FDR ≤ 0.05) and |log2 ratio| ≥ 1 enrichment values are highlighted in **bold**. NA = not applicable.

	vGluT2 [norm. count]	vGAT [norm. count]	Gad67 [norm. count]	vGluT2 vs. vGAT		vGluT2 vs. Gad67	
**Gene**	**Mean ± Stddev**	**Mean ± Stddev**	**Mean ± Stddev**	**log2 Ratio**	**FDR**	**log2 Ratio**	**FDR**
Chrm1	25.6 ± 7.2	30.2 ± 6.1	19.3 ± 7.0	-0.24	9.70E-01	0.39	8.53E-01
Chrm2	5176 ± 811	4818 ± 667	5664 ± 782	0.10	9.13E-01	-0.14	6.30E-01
Chrm3	497 ± 148	1026 ± 160	1183 ± 141	**-1.05**	**2.63E-04**	**-1.27**	**1.17E-07**
Chrm4	89 ± 34	56 ± 28	130.5 ± 19.8	0.66	7.68E-01	-0.57	3.88E-01
Chrm5	177 ± 28	8.8 ± 8.4	23.1 ± 13.1	**4.27**	**3.14E-09**	**2.91**	**7.15E-09**

Table S12 Metabotropic acetylcholine receptors (muscarinic). Significant (FDR ≤ 0.05) and |log2 ratio| ≥ 1 enrichment values are highlighted in **bold**. NA = not applicable.

Serotonin receptors

	vGluT2 [norm. count]	vGAT [norm. count]	Gad67 [norm. count]	vGluT2 vs. vGAT		vGluT2 vs. Gad67	
**Gene**	**Mean ± Stddev**	**Mean ± Stddev**	**Mean ± Stddev**	**log2 Ratio**	**FDR**	**log2 Ratio**	**FDR**
Htr1a	598 ± 103	1503 ± 240	1627 ± 154	**-1.33**	**2.38E-10**	**-1.46**	**6.27E-16**
Htr1b	803 ± 175	529 ± 132	543 ± 75	0.60	1.23E-01	0.55	**4.26E-02**
Htr1d	102 ± 37	214 ± 46	468 ± 102	**-1.08**	**4.51E-02**	**-2.22**	**9.50E-12**
Htr1f	68 ± 28	115 ± 91	60.3 ± 8.1	-0.75	7.50E-01	0.16	8.99E-01
Htr2a	315 ± 56	466 ± 115	510 ± 71	-0.57	2.29E-01	-0.71	**1.03E-02**
Htr2b	0 ± 0	0 ± 0	0 ± 0	0.00	NA	0.00	NA
Htr2c	6685 ± 1060	10081 ± 1679	10828 ± 846	-0.59	**2.50E-02**	-0.71	**3.75E-05**
Htr3a	90.8 ± 21.7	752 ± 124	428 ± 75	**-3.05**	**1.11E-28**	**-2.25**	**1.52E-15**
Htr3b	3.1 ± 5.4	7.7 ± 5.9	7.0 ± 6.7	**-1.29**	NA	**-1.15**	NA
Htr4	197.5 ± 23.6	130.5 ± 23.8	167 ± 60	0.60	3.60E-01	0.23	7.16E-01

Htr5a	525 ± 41	784 ± 59	699 ± 78	-0.58	**3.30E-02**	-0.43	9.91E-02
Htr5b	12.7 ± 10.1	17.2 ± 15.6	23.7 ± 19.8	-0.45	9.68E-01	-0.92	7.10E-01
Htr6	87.4 ± 18.1	66 ± 35	109 ± 35	0.39	8.88E-01	-0.33	6.90E-01
Htr7	804 ± 48	1069 ± 120	828 ± 85	-0.41	1.77E-01	-0.05	8.90E-01

Table S13 Serotonin receptors. Significant (FDR ≤ 0.05) and |log2 ratio| ≥ 1 enrichment values are highlighted in **bold**. NA = not applicable.

Adrenergic receptors

	**vGluT2 [norm. count]**	**vGAT [norm. count]**	**Gad67 [norm. count]**	**vGluT2 vs. vGAT**		**vGluT2 vs. Gad67**	
**Gene**	**Mean ± Stddev**	**Mean ± Stddev**	**Mean ± Stddev**	**log2 Ratio**	**FDR**	**log2 Ratio**	**FDR**
Adra1a	576 ± 61	503 ± 112	595 ± 153	0.19	8.52E-01	-0.06	9.14E-01
Adra1b	498 ± 152	753 ± 98	743 ± 95	-0.60	1.19E-01	-0.59	5.25E-02
Adra1d	101 ± 28	110.0 ± 20.3	117 ± 49	-0.13	9.66E-01	-0.22	8.07E-01
Adra2a	1992 ± 386	942 ± 155	981 ± 189	**1.08**	**6.89E-07**	**1.01**	**1.27E-06**
Adra2b	1.40 ± 2.42	6.0 ± 10.5	26.8 ± 15.0	**-2.05**	NA	**-4.21**	5.84E-02
Adra2c	389 ± 103	329.5 ± 17.7	302 ± 82	0.24	8.29E-01	0.35	4.56E-01
Adrb1	105 ± 46	204 ± 46	162 ± 84	-0.97	1.02E-01	-0.65	3.46E-01
Adrb2	9.0 ± 11.4	3.8 ± 6.7	0 ± 0	**1.21**	NA	**6.25**	NA
Adrb3	12.5 ± 12.5	30.8 ± 22.7	24.5 ± 3.2	**-1.30**	8.12E-01	-0.99	7.08E-01

Table S14 Adrenergic receptors. Significant (FDR ≤ 0.05) and |log2 ratio| ≥ 1 enrichment values are highlighted in **bold**. NA = not applicable.

Dopamine receptors

	**vGluT2 [norm. count]**	**vGAT [norm. count]**	**Gad67 [norm. count]**	**vGluT2 vs. vGAT**		**vGluT2 vs. Gad67**	
**Gene**	**Mean ± Stddev**	**Mean ± Stddev**	**Mean ± Stddev**	**log2 Ratio**	**FDR**	**log2 Ratio**	**FDR**
Drd1	38.0 ± 5.9	48.8 ± 12.7	46 ± 37	-0.36	9.28E-01	-0.29	8.65E-01
Drd2	610 ± 180	961 ± 198	1215 ± 127	-0.66	7.09E-02	**-1.01**	**1.50E-05**
Drd3	9.6 ± 6.7	8.9 ± 4.3	25.0 ± 19.6	0.11	NA	**-1.37**	5.10E-01
Drd4	1.39 ± 2.41	23.5 ± 19.1	9.4 ± 5.4	**-4.02**	2.41E-01	**-2.70**	NA
Drd5	221 ± 39	135 ± 100	209 ± 35	0.71	5.10E-01	0.07	9.17E-01

Table S15 Dopamine receptors. Significant (FDR ≤ 0.05) and |log2 ratio| ≥ 1 enrichment values are highlighted in **bold**. NA = not applicable.

Histamine receptors

	**vGluT2 [norm. count]**	**vGAT [norm. count]**	**Gad67 [norm. count]**	**vGluT2 vs. vGAT**		**vGluT2 vs. Gad67**	
**Gene**	**Mean ± Stddev**	**Mean ± Stddev**	**Mean ± Stddev**	**log2 Ratio**	**FDR**	**log2 Ratio**	**FDR**
Hrh1	39.1 ± 9.8	35.2 ± 21.0	73 ± 38	0.14	9.81E-01	-0.92	3.32E-01
Hrh2	80.1 ± 21.4	77 ± 36	68.8 ± 7.8	0.05	9.92E-01	0.21	8.37E-01
Hrh3	942 ± 181	1428 ± 79	1523 ± 94	-0.60	**1.36E-02**	-0.71	**4.04E-04**
Hrh4	0 ± 0	0 ± 0	0 ± 0	0.00	NA	0.00	NA

Table S16 Histamine receptors. Significant (FDR ≤ 0.05) and |log2 ratio| ≥ 1 enrichment values are highlighted in **bold**. NA = not applicable.
